# Reporting to parents on children’s exposures to asthma triggers in low-income and public housing, an interview-based case study of ethics, environmental literacy, individual action, and public health benefits

**DOI:** 10.1186/s12940-018-0395-9

**Published:** 2018-05-21

**Authors:** Laura J. Perovich, Jennifer Liss Ohayon, Elicia Mayuri Cousins, Rachel Morello-Frosch, Phil Brown, Gary Adamkiewicz, Julia Green Brody

**Affiliations:** 1 MIT Media Lab, Massachusetts Institute of Technology, Cambridge, MA USA; 2 Silent Spring Institute, Newton, MA USA; 3 Social Science Environmental Health Research Institute, Northeastern University, Boston, MA USA; 4 Department of Sociology and Anthropology and Social Science Environmental Health Research Institute, Northeastern University, Boston, MA USA; 5 Department of Environmental Science, Policy and Management and School of Public Health, University of California, Berkeley, Berkeley, CA USA; 6 Harvard T.H. Chan School of Public Health, Harvard University, Boston, MA USA

**Keywords:** Return of results, Environmental health literacy, Asthma, Research ethics, Biomonitoring, Community-based participatory research, Exposure assessment, Risk communication

## Abstract

**Background:**

Emerging evidence about the effects of endocrine disruptors on asthma symptoms suggests new opportunities to reduce asthma by changing personal environments. Right-to-know ethics supports returning personal results for these chemicals to participants, so they can make decisions to reduce exposures. Yet researchers and institutional review boards have been reluctant to approve results reports in low-income communities, which are disproportionately affected by asthma. Concerns include limited literacy, lack of resources to reduce exposures, co-occurring stressors, and lack of models for effective reporting. To better understand the ethical and public health implications of returning personal results in low-income communities, we investigated parents’ experiences of learning their children’s environmental chemical and biomonitoring results in the Green Housing Study of asthma.

**Methods:**

The Green Housing Study measured indoor chemical exposures, allergens, and children’s asthma symptoms in “green”-renovated public housing and control sites in metro-Boston and Cincinnati in 2011–2013. We developed reports for parents of children in the study, including results for their child and community. We observed community meetings where results were reported, and metro-Boston residents participated in semi-structured interviews in 2015 about their report-back experience. Interviews were systematically coded and analyzed.

**Results:**

Report-back was positively received, contributed to greater understanding, built trust between researchers and participants, and facilitated action to improve health. Sampling visits and community meetings also contributed to creating a positive study experience for participants. Participants were able to make changes in their homes, such as altering product use and habits that may reduce asthma symptoms, though some faced roadblocks from family members. Participants also gained access to medical resources, though some felt that clinicians were not responsive. Participants wanted larger scale change from government or industry and wanted researchers to leverage study results to achieve change.

**Conclusions:**

Report-back on environmental chemical exposures in low-income communities can enhance research benefits by engaging residents with personally relevant information that informs and motivates actions to reduce exposure to asthma triggers. Ethical practices in research should support deliberative report-back in vulnerable communities.

**Electronic supplementary material:**

The online version of this article (10.1186/s12940-018-0395-9) contains supplementary material, which is available to authorized users.

## Background

Personalized information about chemical exposures may be an effective way to motivate and inform parents, so they can reduce asthma triggers in their homes. Asthma affects 6.2 million children in the US [1], and asthma rates for non-Hispanic black children and Puerto Rican children are almost twice as high as rates for non-Hispanic white children [2]. More than 1 in 10 children below the federal poverty level has asthma [2]. Effects include decreased educational success, decreased overall health, and increased emergency room visits, which reduce family income when parents or guardians miss work and limit time to care for other family members [3–6]. Asthma can be triggered by environmental agents including air pollutants and allergens in the home that may be reduced by the choices of building materials, ventilation, and housing management practices in green buildings [7, 8]. A study in Boston public housing found that children in green renovated units had fewer asthma symptoms, asthma attacks, hospital visits, and missed school days than in traditional unrenovated homes [8, 9]. Increasing evidence indicates that endocrine disrupting compounds, such as phthalates (e.g., DEHP, benzylbutylphthalate) and phenolic compounds (e.g., triclosan, bisphenol A, and parabens), can exacerbate asthma symptoms [10–17]. Reporting to parents in studies of these chemical exposures may enable them to make personal changes and advocate for institutional change to reduce asthma triggers.

In the past, reporting personal chemical-exposure results to study participants was mostly limited to compounds with established clinical guidelines, like lead, but as newer practices, based on right-to-know ethics, are becoming more common, report-back extends to endocrine disrupting chemicals with less established health effects [18–27]. When reports include contextual information about health, uncertainties, and opportunities for exposure reduction, studies find that report-back leads to greater understanding, motivates action, and does not cause excessive worry [18, 24, 28, 29]. Still, researchers and IRBs often remain hesitant to report results in low-income communities, citing limited literacy, language barriers, lack of resources to reduce exposures, adding worries and “action items” to an already-stressed group, and limited prior models for reporting back [25]. Yet low-income communities may particularly benefit, because report-back contributes to environmental health literacy, discovery of local exposure sources, and a respectful, transparent research context [24, 26, 29, 30]. The Green Housing Study (GHS) illustrates these issues. GHS is funded by the U.S. Centers for Disease Control and Prevention (US CDC), Department of Housing and Urban Development, and National Center for Healthy Housing to evaluate effects of home environments on children with asthma living in public housing. Participating families have limited formal education, are stressed by a child with asthma, and have constrained capacity to influence the conditions of public housing or low-cost rental housing. At the same time, this setting creates opportunities to build capacity among residents, improve their health, engage housing decision-makers, and address environmental health with local clinicians. To inform best practices for results communication in low-income communities, we conducted a case study of the experiences of low-income housing residents who were part of the GHS.

## Methods

The Green Housing Study is investigating how “green” renovation in low-income housing affects indoor air quality and asthma morbidity in children. From 2011 to 2013, researchers from Harvard School of Public Health, University of Cincinnati, and Silent Spring Institute collected data on indoor environmental chemicals, allergens, and children’s asthma [31]. The study was conducted in renovated and control low-income and public housing in metro-Boston (Lowell, Lawrence, Old Colony, Castle Square) and Cincinnati. Renovations focused on energy efficiency, with some Boston sites also adopting integrated pest management and removing vinyl flooring. The primary aim was to investigate effects of energy-saving buildings, which modify ventilation, on air quality and health.

The GHS recruited children aged 7 to 12 with doctor-diagnosed asthma who lived in public housing developments and other nearby low-income units [31]. The study team recruited participants who lived in renovated public housing through community meetings led by GHS staff, distributing fliers, and door-to-door visits [31]. Interested participants could get more information and join the study during community meetings or by contacting study staff by telephone. Recruitment of control homes at comparable sites in the broader Boston community occurred through fliers at health centers, hospitals, community-based organizations, community programs, and after-school programs, and recruitment letters sent by the Boston Housing Authority to select individuals living in Boston public housing. Recruitment letters were sent in three languages and instructed interested individuals to contact the Green Housing Study team by telephone for additional information.

Researchers conducted three or four home visits over one year, collecting household dust, indoor and outdoor air, children’s urine and blood, and assessments of airway inflammation and lung function [31–35]. Parents responded to questionnaires about demographics, home characteristics, and the children’s health. Over one hundred environmental chemicals were analyzed, including pesticides, flame retardants, fragrances, polycyclic aromatic hydrocarbons (PAHS, products of combustion), polychlorinated biphenyls (PCBs), chemical sunscreen (benzophenone-3), parabens, phthalates, and triclosan. Many of these measurements are novel and household exposure to a few of these chemicals has not been previously reported. Spot and morning void urine samples were taken at the home visit, frozen on site, collected when the sampling equipment was retrieved from the home (up to 5 days), and stored at -20 °C until shipment to the lab for analysis. SVOC indoor air samples were collected from the main living area using URG personal pesticide sampling cartridges (University Research Glassware; Chapel Hill, NC) [33, 34] and surface wipe samples were collected from the kitchen floor using the protocols from the American Healthy Homes Survey [32]. Indoor air and surface wipe samples were analyzed via GC/MS at Southwest Research Institute and first void morning urine samples were analyzed via high performance liquid chromatography tandem mass spectrometry (HPLC-MS/MS) at the CDC Environmental Health Laboratory [36, 37]. When first morning void samples were not available (*n* = 13), spot urine samples were analyzed.

Select participants were recruited for semi-structured interviews about their experience after receiving their study results. Participants from Old Colony (*n* = 24 eligible participants) were informed about report-back interviews during community meetings and participants from Old Colony and Lowell/Lawrence (*n* = 6 eligible participants) were recruited for these interviews by phone call in the year following the community meetings. Interviews were conducted in the order that participants were reached by phone.

### Report-back methods

**Fig. 1:**
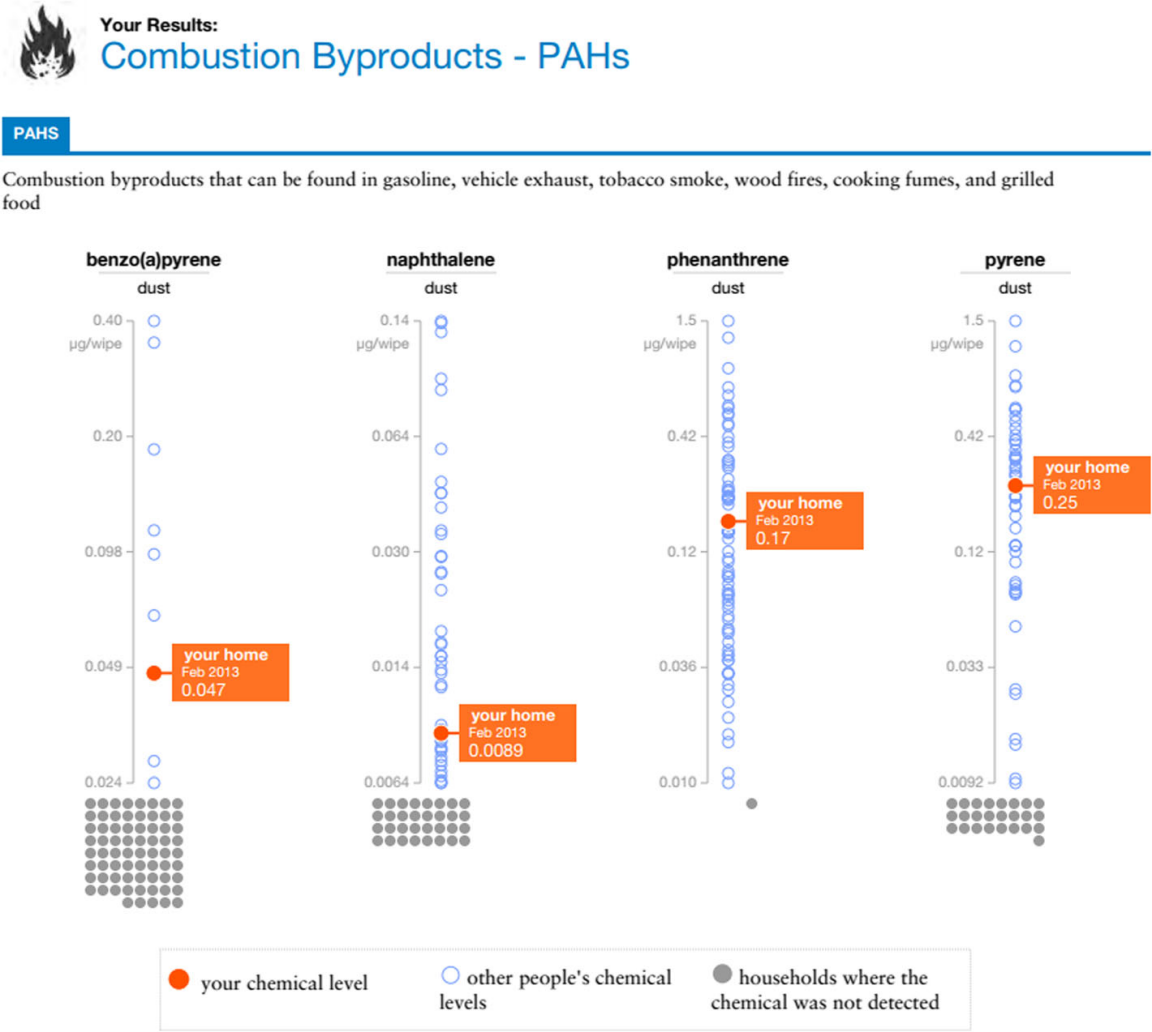
An example graph from the second personalized report-back packet received by study participants

Parents of the children in the study received two report-back packets, one during a home sampling visit and one by mail or at a community meeting. Reports integrated input by GHS and the Personal Report-back Ethics (PERE) Study teams, based on best practices in health communication, testing of prototypes with residents of the Boston study neighborhood, and previous research on report-back [18, 22]. Reports included personalized results graphs, text summaries, and information about exposure reduction, chemical sources, and health. The second report, which included results for environmental chemicals, was personalized using DERBI, the Digital Exposure Report-back Interface [22]. An example results graph from the second report is shown in Fig. 1 and a full report is shown in Additional file 1. Boston participants also received personalized data shirts with a visual representation of selected chemical results [38]. Health-related information in the reports focused on asthma and included brief information about other health impacts. For example, the overall study results section of the report says:

We found fragrance chemicals – which are avoidable asthma triggers – in every home. Most of the homes also had antibacterial chemicals, another avoidable asthma trigger. These chemicals have been linked to worsening asthma symptoms.

Open community meetings for participants and the public were held in Cincinnati and Boston. Meetings and written reports were translated based on languages spoken in the community (Cantonese, Mandarin, or Spanish). Among 44 Massachusetts participants who completed the study, 23 participants who attended a community meeting received their second result packet at that meeting and 21 participants who did not attend a community meeting received their second results packet by mail (see Table 1). The Cincinnati meeting was well-attended by the public, though no study participants attended. Researchers attended all community meetings and made notes of their observations.

**Table 1:** Study participation and eligibility for meetings and interviews. Three out of four sites had community meetings, and participants from two sites were recruited for report-back interviews

	Participants Engaged in Study Activities (N)			
Site	Completed GHS sampling	Attended community meeting	Recruited for interview	Completed interview
Cincinnati	30	0	0	N/A
Lawrence/Lowell	6	N/A	6	3
Castle Square	14	14	0	N/A
Old Colony/other Boston	24	9	24	7

**Fig. 2:**
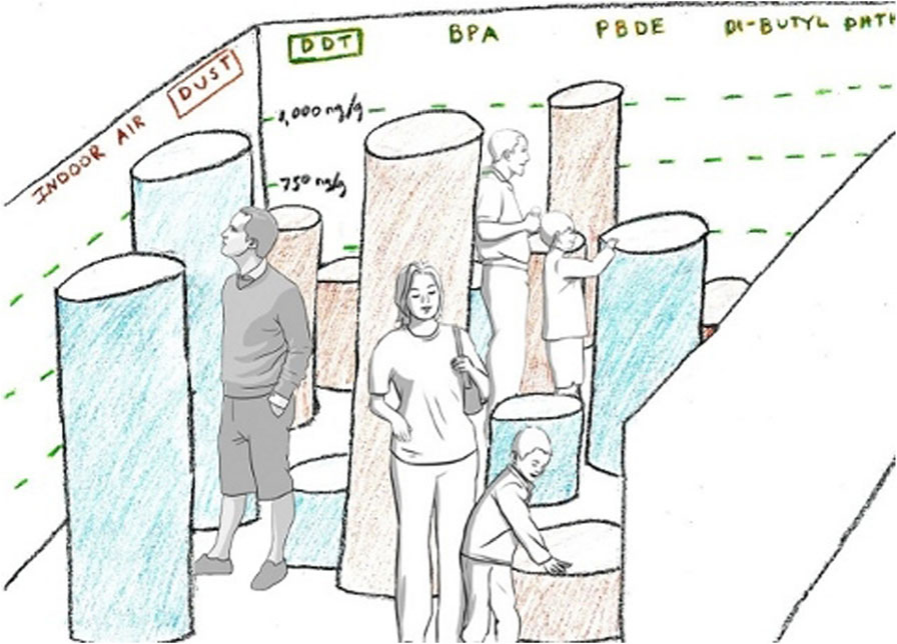
A concept sketch of BigBarChart, a data physicalization that was demonstrated at the Cincinnati community meeting

**Fig. 3:**
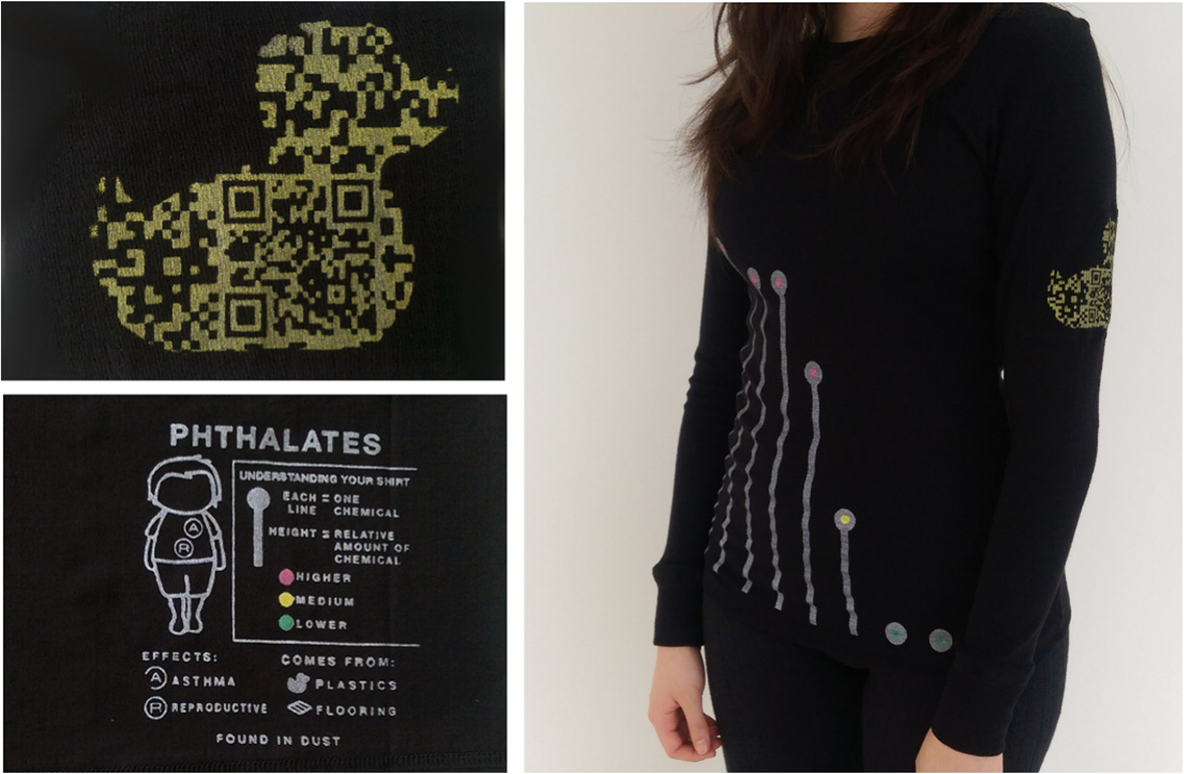
Personalized data shirts were given to participants in the Boston Green Housing Study sites as part of their report-back packet. A shirt shows the relative amount of phthalates found in house dust for each individual compared to other participants in the study

The Cincinnati community meeting also provided an opportunity for the study team to demonstrate two prototype data physicalizations [39, 40]: BigBarChart and Dressed in Data [41]. Data physicalization is an emerging field that expands on data visualization research to create physical objects that display data in the real world, instead of on paper or a screen. BigBarChart (Fig. 2) is a human-sized, three-dimensional bar chart that participants can interact with using tangible interfaces to explore the community’s chemical exposure data. Each bar is made from a modified laundry hamper and can change its height and color to represent different quantitative (e.g. chemical amount) and qualitative (e.g. city) variables as the participants explore the dataset. Dressed in Data (Fig. 3) is a set of shirts that show individual participants’ exposure data for one class of compounds (phthalates). The shirt has three parts: a simplified line plot on the front of the shirt shows the relative amount of each phthalate found in the participant’s dust compared to other people in the study, a tag inside the shirt gives additional information about the sources and health risks, and a duck-shaped QR code on the sleeve links to more detailed information.

### Report-back interviews and analysis

After parents received their reports, the Boston GHS collaborators recruited 10 Old Colony and Lowell participants for semi-structured interviews based on the methods of previous studies [21, 30]. Interviews lasted about one hour and were conducted in person by trained staff and recorded with permission.

The semi-structured interview protocol had a sequence of questions and probes that systematically covered the study themes while allowing the participant to freely add information. Interviews began by asking participants about their overall experience in the study and during sample collection. Questions asked about participants’ thoughts or feelings when reading their study reports, the usefulness of elements in the report, understandings about the results, whether they found the report-back information surprising, and individual or collective actions taken or planned to reduce exposures. Questions asked whether participants had spoken to others about their reports and about those conversations. Interviews also invited participants to give advice to researchers (see Additional file 1 for interview guide).

Transcripts were analyzed and discussed by three members of the research team to develop codes based on interview questions, as well as broader conceptual themes reflected in the protocol. Important passages were identified from the interview transcripts based on these codes and summarized in order to draw out overarching patterns within and across the themes and participants. Four interview transcripts were analyzed by two researchers to compare code application and ensure inter-coder reliability, and one team member analyzed the remaining interviews after the coding scheme was well-developed. Roughly 15 revised themes were identified by researchers in response to patterns in interviews and on the ground knowledge of the study and were culled and finalized collaboratively based on strength of evidence and significance to report-back researchers. Interviews were reassessed by 1–2 researchers, based on the new themes, to determine frequency, and select relevant quotes.

## Results

Ten Boston-area mothers completed interviews, 7 in English, 2 in Spanish, and 1 in Mandarin. The interviews consistently revealed that these mothers living in low-income housing and parenting a child with asthma appreciated receiving their study results and used the information. They made efforts to reduce their child’s asthma symptoms by altering product use and habits. Sampling visits, report-back, and community meetings all contributed to greater understanding and also built trust between researchers and participants, which improved the study experience. Some participants leveraged the study to advocate for better medical care, yet some felt that clinicians were not responsive. Some participants also faced roadblocks from family members who did not want to adopt healthy changes, and some wanted assistance from researchers to facilitate institutional change. We discuss six main themes that emerged from the interview analysis and summarize the key supporting points in Table 2.

**Table 2:** Frequency of key participant experiences by theme

Theme	Coded Attribute	Percent of Participants (*N* = 10)
Theme 1	Joined study to access information or test otherwise unavailable	100%
Theme 2	Cited specific individual changes planned or made in response to the study	80%
Theme 3	Used study results to access additional medical or government resources	40%
Theme 4	Shared or expressed intent to share study information with family or local community	90%
Theme 5	Cited positive personal attributes of the study staff	80%
Theme 6	Cited desire for community-level change from the government or industry	100%
Theme 6	Cited specific action planned or made to prompt community-level change	0%
Overall	Expressed regret about receiving study results	0%
Overall	Cited benefits of report-back	100%

### Theme 1: Participants were motivated to learn about home exposures and their families’ health

All participants said they were motivated to join the study to access information or medical tests that were otherwise unavailable to them. Specifically, they wanted to learn more about their child’s asthma—the investigation’s central theme—as well as skin conditions, allergies, and general health. Many hoped to use this information to improve their child’s health:

I was not clear why [my child] was getting sick all the time. So I want to go deeper to find out what he was allergic to... I went to a place to have an allergy test and they just start giving him medications... I want to know more why you’re giving all these meds. (P02).

### Theme 2: Participants used the results to make positive changes

All participants cited benefits of their reports, and almost all cited specific changes they made or intended to make in response to the study, mainly by using alternative products, minimizing certain products, and changing household habits:

For example I thought that using spray chemicals to kill cockroaches was the most effective way to kill them, but now I have replaced it with cockroach sticky traps, decreasing the use of the spray chemicals. And moreover, after I learned from this research that products with fragrances can increase the chemical concentration in the house, I have decreased the use of products with fragrances. Take shower gel as an example, I now understand that those without fragrances would be better. (P05).

**Fig. 4:**
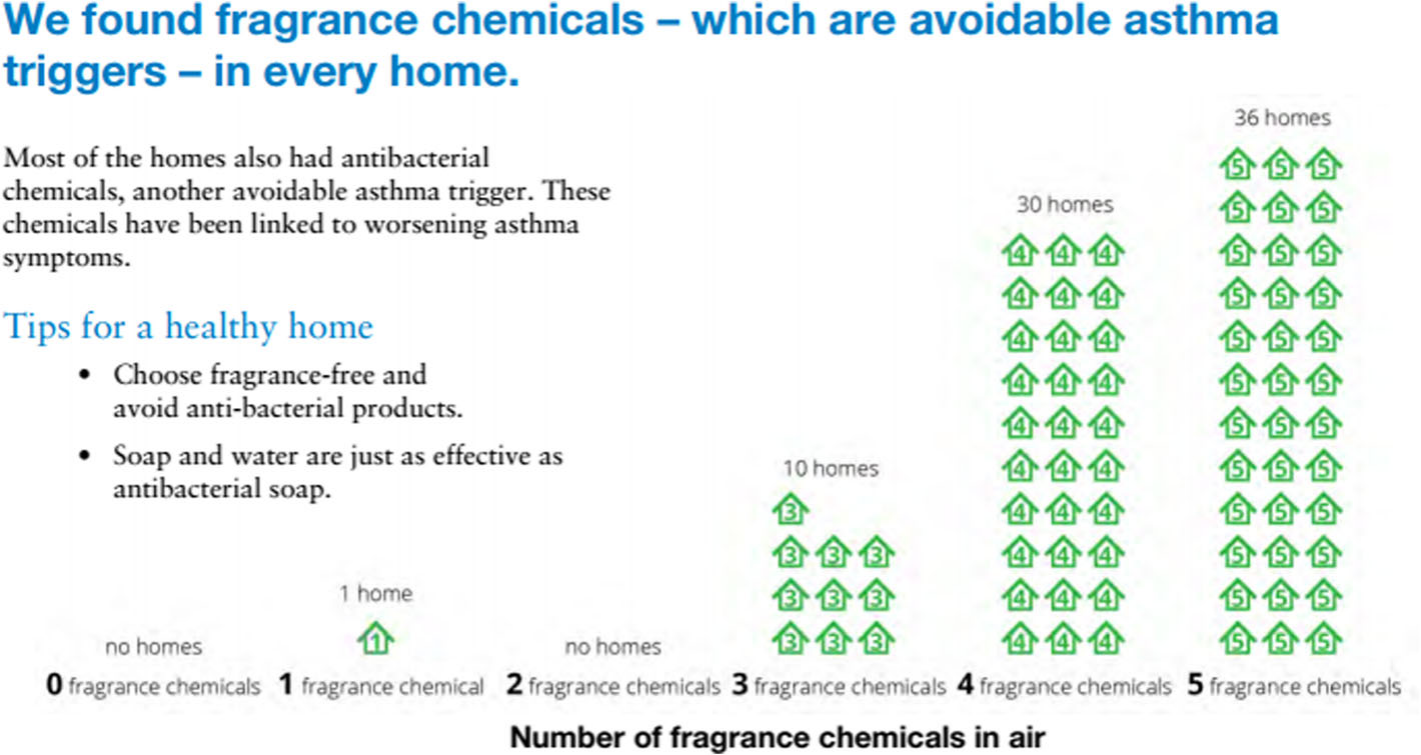
This pictograph of study-wide results shows that fragrance use was ubiquitous in homes. The text links the results to actions that can reduce asthma symptoms for children in the study

**Fig. 5:**
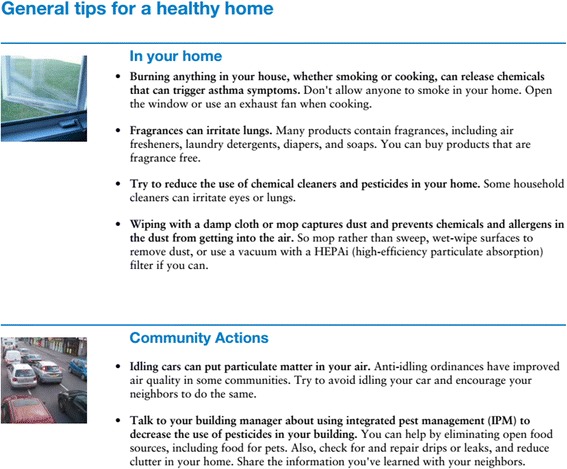
The report included tips for reducing chemical exposures related to asthma. Almost all participants made changes inside their home to reduce exposures; researchers could do more to support participants seeking community action

Many participants cited fragrance as a particular target for change, which is significant, because avoiding fragrance was a main message in the reports (see Fig. 4). Participants also described eliminating nearby smoking, cleaning when children are out of the home, or covering pillows. The ability of participants to articulate specific actions to reduce exposures to hormone disruptors (see Fig. 5) and understand the relevance to their child’s asthma shows how report-back can enhance environmental health literacy [29, 42].

### Theme 3: Participants leveraged study results to access medical or government resources but with mixed success

Results reports provided leverage for participants in low-income housing to access medical resources and government programs. Two participants brought the report to their children’s doctors to get bed coverings:

We talk [with her doctor] about the covering of her beds and her pillows. And matter of fact, they were able to give me a prescription and MassHealth [Medicaid] paid for all the covering of her bed. (P06).

Participants also used the study to access specialists and get inhalers or other medical treatment (P06, P03, P02).

However, other participants who were eager to talk to their healthcare providers about their child’s results remarked that their physicians seemed uninterested or unversed in environmental health. For example, one said her doctor focused on prescribing asthma medication and allergy shots rather than discussing strategies to improve her family’s indoor environment.

### Theme 4: Participants faced successes and challenges in creating change in families and communities

Similarly, some participants worked to motivate their families and communities to make changes based on study results. They shared information with family and social circles, often with preference to individuals affected by asthma. They used research results to start conversations about healthier habits. However, promoting changes could be challenging:

From the report I learned that a lot of chemicals can be harmful to your health, a lot of things that I need to improve on. But my father and son do not seem to take this issue seriously, and I feel like I can’t make a major change. But for the future, I think the report can help them understand the factors that lead to their asthma. (P05).

Moreover, participants noted language and cultural barriers to sharing information with neighbors in communities of mixed Hispanic and Chinese families (P05).

### Theme 5: Participants gained understanding and built trust with researchers throughout the study process

Interviews revealed that environmental health literacy was developed through both the report-back and other aspects of the study process, including at-home sampling visits, questionnaires, and community meetings. Participants recognized that the sampling and surveys were “all a learning process” (P03)*.* Completing questionnaires raised the idea that topics in the questions might be associated with health risks. One participant said she was “surprised to find out about some questions that I wasn’t expecting to be asked” (P07), such as about using an exhaust fan while cooking. Participants informally collaborated with researchers during sampling visits to consider steps to improve health—for example, one invested in an air conditioner after discussing with a researcher the frequency with which individuals smoked outside her window (P03).

Community meetings provided another opportunity to discuss results and led to additional insights. For example, two participants with high PAHs in indoor air found each other during the informal conversation time and together approached a researcher. Conversation revealed that one family was frequently burning incense, while the other had a broken kitchen fan and frequent high-temperature wok cooking contributed to high levels of smoke.

Some participants emphasized that the study would have felt intrusive without the report-back process. As one stated:

In the beginning I didn’t like it… You get that feeling like when somebody’s intruding….But then, after seeing the result [at the mid-study visit]... I felt more comfortable because this was for the benefit of my daughter and her asthma. (P06).

Several participants alluded to how report-back added reciprocity between researcher and participant: “[I give] information to you guys who are doing the study and then get some information back for my own interest” (P09).

Most participants cited personal attributes of the researchers as facilitating their participation in a study that could otherwise be uncomfortable. They appreciated that researchers were “friendly” (P02), “professional” (P04), “patient” (P07), “flexible” (P10), and “trusted” (P02). Researcher attributes helped to manage cultural barriers:

you guys make me feel comfortable and, and it’s hard for you to open the door to strangers...Well, I’m Spanish, you know. I don’t want people to come to my house and check to see I have dust in my house, if my house is dirty… (P02).

### Theme 6: Participants want community-level change

While many participants cite individual actions to reduce asthma triggers, few cite concrete possibilities for collective action and none reported taking such action. Participants believe that companies and the government are unlikely to institute changes. In response to a question about company practices, one participant stated:

oh boy. I don’t know what to say about that because if they know it’s bad for the environment, they know it’s bad for people that use them, but it’s about money, they’re not gonna do any change. (P07).

Despite their pessimism, participants still hope for change. As one indicated:

[The government] needs to change those rules because it’s not only my child, you can see a lot of children with asthma, a lot. (P01).

Participants were interested in knowing what researchers would do with the data and some said they wanted the researchers to shape institutional changes. They asked whether data would be shared with hospitals, doctors, or others with decision-making power.

### Results do not cause excessive worry to participants

In addition to observing participant responses, we also note what they did *not* say. During interviews, participants were asked about their thoughts and feelings upon receiving their study results, whether they were surprised by anything in their results, and whether they were glad to learn their personal results. Consistent with findings in other report-back studies, no participants reported excess worry, anxiety, or distress at receiving their results. Some participants reported that they expected their exposures to be high which made their results less surprising:

I predict that the chemical concentration level would be pretty high in my home, so I wasn’t too surprised. But I was still surprised that the levels were pretty high. (P05).

Most participants were glad to receive their results, sometimes because the results provided important feedback that participants could act on:

Yes. Now I know everything, ... what I was doing wrong or what I should do, be doing better, I’m glad to find out with that. We have some, not issues, we have things we can work out with them make a little better. (P07).

Interactions with participants during home visits, interviews, and community meetings also showed that participants asked questions and were engaged and curious and not overly burdened by the study results.

## Discussion

This case study shows that reporting personal results for chemical exposures can be a positive component of ethical research practice for residents of low-income and public housing, because it provides useable information to address health disparities. Residents learned about actions to reduce their child’s asthma symptoms, increased their broader understanding of environmental health, and leveraged their results to access medical care. Based on this case study, researchers and IRBs can feel more confident that report-back practices can be adopted effectively among public and low-income housing residents.

Communication between participants and researchers throughout the study also increased trust, an important outcome, because it helps to remedy the history of distrust resulting from past research abuses in communities of color and indigenous communities. For example, in a study of lead paint remediation in low-income housing, researchers failed to inform study participants that their children’s blood lead levels were rising in one arm of the study and did not promptly end that intervention [43, 44]. This ethical failure and others have understandably made communities of color reluctant to participate in research, which, in turn, limits the generation of knowledge that could benefit them. In this context, report-back of personal exposures to environmental chemicals is particularly valuable. Further, receiving information on how to reduce these exposures can transform the researcher-participant dynamic to a more reciprocal relationship, in which participants feel they are benefitting from the data that they are providing to investigators.

Earlier studies of report-back in environmental health research have similarly observed positive experiences and contributions to personal and social change [18, 20, 23, 25, 45, 46]. However, IRBs and researchers have been concerned that in communities with multiple social and economic stressors and restricted options, report-back might add emotional burden without increasing health efficacy. Our study is small, which limits generalizability; however, our interviews showed that report-back can motivate participant engagement with personally relevant health issues, even in communities where time and resources are scarce. Similar to prior studies [18–21, 23, 45], report-back in the GHS was a desired component of the research, and we did not observe undue worry or feelings of unwanted burden among study participants in response to their child’s results and health messages.

We expect that the success of report-back in this study is partly due to tailoring information to the community context. Since many GHS participants were motivated to join the study because of their child’s asthma, the report-back was designed to include accessible ways to improve asthma symptoms, such as reducing fragrance use. Input from community leaders and usability testing of prototypes helped to shape the format and content to local needs and cultural contexts.

Our study also revealed ideas for improvement. For example, some GHS participants leveraged results to access medical resources. In the future, results reports could include a short briefing written for physicians, which participants could bring to their doctors. Similarly, participants faced barriers from family and others unwilling to change habits (e.g. smoking inside) and from practices by building management (e.g. pesticide spraying). The study could enhance participants’ efficacy by addressing these barriers. For example, researchers could educate family members and other residents, and participate in meetings with the housing management company.

Many GHS participants wanted changes by industry or government, but did not find ways to act on these issues. Researchers could help participants develop advocacy roles or connect with organizations that are already working to improve chemical regulation or housing conditions, such as the Green Building Council. A recent review of environmental health literacy studies suggests that increasing community engagement throughout the course of a study and putting additional resources towards increasing self-efficacy can help study participants expand from individual change to community-level change [29]. Because individuals who receive personal reports become more informed and motivated, they are poised to become change agents [30]. Researchers can increase the public health impact of their findings by facilitating those efforts, although we acknowledge that these activities take time and require additional skills. Developing ethical and effective methods for researchers to support participants’ desires for collective action remains an area for further exploration for report-back and environmental health literacy studies [29].

Future studies can build on this GHS case study by conducting data reporting in low-income and other vulnerable communities, and by adapting our report-back model in the particular context of their study participants. As researchers expand the practice of reporting-back in many different communities with diverse populations, new research about report-back in multiple settings can expand our knowledge about people’s experience receiving their data and provide a range of report-back models for researchers. Beta testing, field testing, and language testing of report-back materials in communities prior to reporting is an important step in effective report-back.

Researchers can also work to increase the opportunity for participants to take action on their results by expanding engagement with the participant’s immediate circles (e.g. family, neighbors, doctors and nurses) and local or national advocacy groups. Researchers could empower participants to connect with local or national groups working on related issues by including literature tables at community meetings or inviting advocates to attend meetings. They could also develop new kinds of reports for different audiences, such as doctors, public housing managers, or family members of the participant. The gender dynamics of report-back may be a particularly relevant area for study, as we found that women were the primary report-back recipients and one reported that male family members resisted changes in the home made in response to the results. In addition, some authors note that when exposure reduction requires informed consumer choice, the burden of implementation falls on women, because they are the primary shoppers and family caregivers [47]. Further research could explore the nature of the gender dynamic in report-back, particularly around home-based exposures, and design approaches that increase family engagement.

Finally, future work can explore alternative tools and platforms for reporting data and explore whether report-back leads to behavior change that impacts health. Digital interfaces for report-back may be less resource-intensive for researchers to deploy and may make it easier for participants to navigate through large amounts of data. New methods of data display, such as the data physicalizations Dressed in Data and BigBarChart prototyped in this study, may create novel or memorable experiences for participants interacting with their data. Cohort studies or other longer-term studies could take a deeper look at the behavior changes participants have described to us and provide more information about the influence of report-back on behavioral changes and their impact on health.

### Limitations

The small number of one-on-one interviews in this case study were limited to metro-Boston and are not representative of all low-income housing communities. Because of the long report-back timeline, we were unable to reach some eligible Boston-area participants to recruit them for interviews, because they apparently relocated. Communications from GHS led Castle Square families to think their participation in the study was concluded when they received reports, so we were not able to recruit them for interviews. In Cincinnati, we attended the community meeting but did not interview individuals due to travel constraints. However, this case study offers unique perspective on the report-back experience in low-income housing and sets the stage for further study of report-back in vulnerable populations.

## Conclusions

This study demonstrates that reporting back individual results for chemical exposures in public housing and other low-income housing can build environmental health literacy, stimulate behavior change, and encourage engagement and trust in research. Returning individual results has become an accepted ethical practice in most research settings [48], and concerns that report-back in low-income communities would be unwelcome and burdensome were not supported. Report-back is an important ethical practice, as it enables participants to benefit directly by receiving actionable health information in return for the data they provide. This case study shows that report-back can enhance the public health benefits for participants living in low-income housing and that IRB policies should encourage carefully designed and deliberative report-back protocols in vulnerable communities.

## Additional file

Additional file 1: (1) Report-back packet: An example GHS personal report is attached and also available at https://silentspring.org/sites/default/files/greenhousingstudy-reportback.pdf. (2) Semi-structured interview guiding questions. (PDF 839 kb)

## Abbreviations

CDC: Centers for Disease Control and Prevention
DERBI: Digital Exposure Report-back Interface
GHS: Green Housing Study
IRB: Institutional Review Board
P0#: Participant number
PAH: Polycyclic aromatic hydrocarbon (products of combustion)
PCB: Polychlorinated biphenyl
PERE: Personal Report-back Ethics
US: United States
