# Supplementary material for: Reporting to parents on children’s exposures to asthma triggers in low-income and public housing, an interview-based case study of ethics, environmental literacy, individual action, and public health benefits
Source: Environ Health. 2018 May 21;17:48. doi: 10.1186/s12940-018-0395-9 (PMC5963109; doi:10.1186/s12940-018-0395-9)
Supplement: Supplementary file 1 — (1) Report-back packet: An example GHS personal report is attached and also available at https://silentspring.org/sites/default/files/greenhousingstudy-reportback.pdf. (2) Semi-structured interview guiding questions. (PDF 839 kb) [file 12940_2018_395_MOESM1_ESM.pdf]

Additional file 1

"Reporting to parents on children's exposures to asthma triggers in low-income and public housing, an interview-based case study of ethics, environmental literacy, individual action, and public health benefits"

Perovich, LJ; Ohayon, J; Cousins, EM; Morello-Frosch, R; Brown, P; Adamkiewicz, G; Brody, JG

2018

Contents:

(1) Example GHS report-back packet (also available at https://silentspring.org/sites/default/files/greenhousingstudy-reportback.pdf)

(2) Semi-structured interview guiding questions

(1) Example GHS report-back packet

(also available at https://silentspring.org/sites/default/files/greenhousingstudy-reportback.pdf)

Green Housing Study

Your Family's Report

2015

Table of Contents

Introduction 5

Summary of What We Found 6

Overall Study Results 8

How to Read Your Graphs 10

Asthma 12

Pesticides 18

Flame Retardants 22

Fragrance Chemicals 30

Combustion Byproducts - PAHs 34

Banned Industrial Chemicals - PCBs 38

Personal Care Product Chemicals 42

Plastics Chemicals 48

Appendix 54

For more information, contact us by phone at 617-332-4288 or email at GHS@silentspring.org

© 2015 Silent Spring Institute.
This work is licensed under a Creative Commons Attribution 4.0 International License.

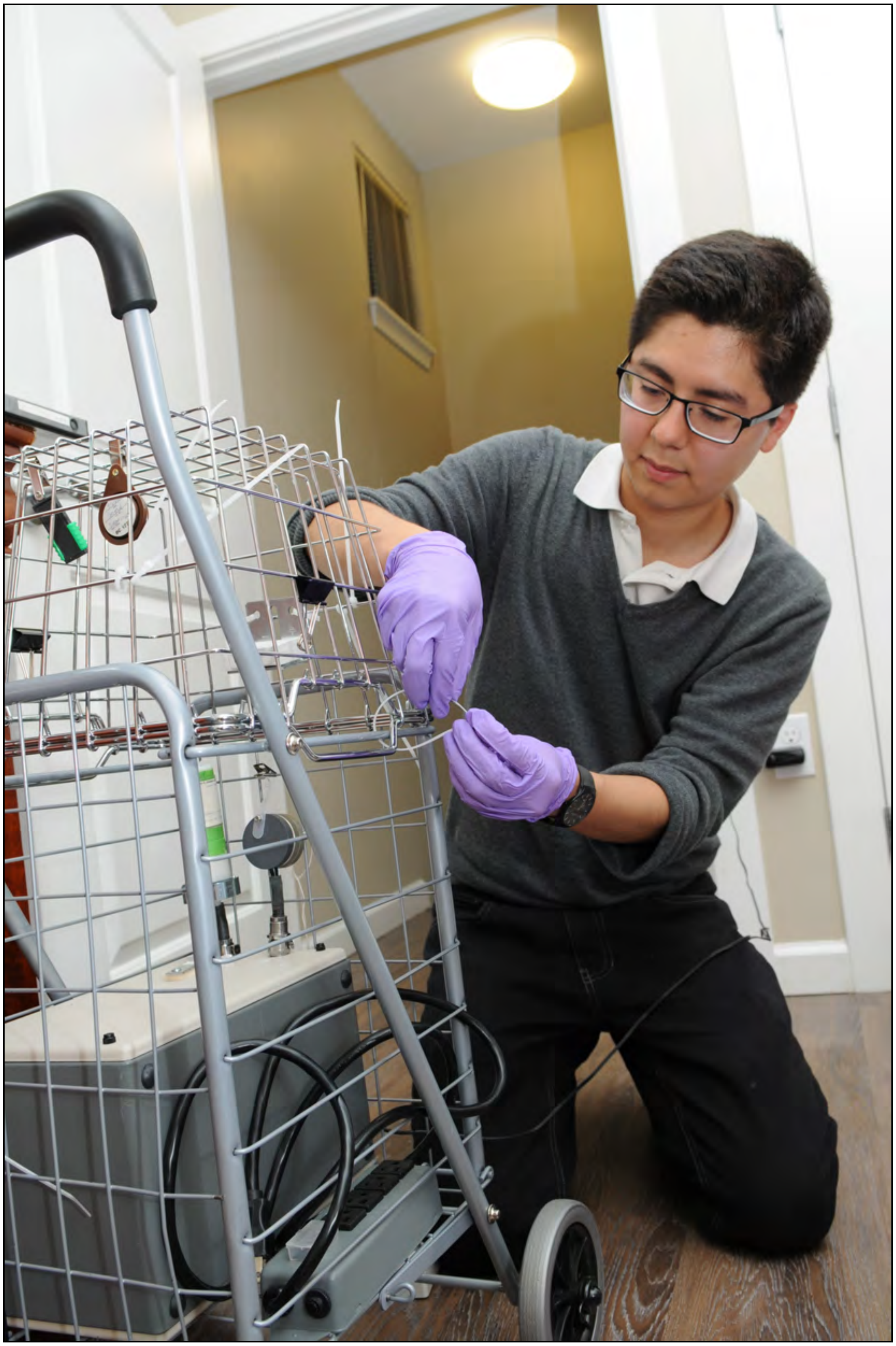

Researchers collected household air and dust and children's urine samples to learn more about how home environments can affect asthma.

Introduction

The number of children with asthma has gone up in the last few years. Some people think that allergens and chemicals that are common inside homes are adding to the problem.

We wanted to learn more about some of the chemicals in pesticides (used to kill bugs or weeds) and everyday things – from shampoo and soap to couches and shower curtains.

This study

- We went to 94 homes, including yours, to measure the levels of 75 chemicals.
- We measured household air and dust and children's urine samples.
- We looked for chemicals that might affect breathing and asthma as well as other health problems.

The study results will help us learn how common chemicals can affect asthma.

This report will tell you what we found in your home and your child's urine sample. You will be able to see whether your results are higher or lower than other people's. And we will tell you how to lower the levels of chemicals in your home.

We will tell you if your results are above a health guide. But for most of the chemicals, scientists don't know enough yet to say how they could affect health for your family. We are studying these chemicals to learn more. We still want to show you your results because there are often things you can do to reduce your exposure.

© 2015 Silent Spring Institute.
This work is licensed under a Creative Commons Attribution 4.0 International License.

Summary of What We Found

Your home had one of the highest levels of a **personal care product**. These chemicals come from products like soap, shampoo, and deodorant.

See page 42

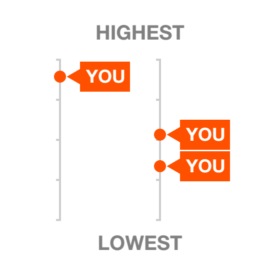

Your samples had higher levels of **combustion byproducts** than most others in the study. These chemicals can cause breathing problems.

See page 34

Your samples had a higher level of a **flame retardant** than most others in the study. These chemicals can come from furniture foam.

See page 22

Other Chemicals

- Your samples had lower levels than most people for fragrance chemicals.

See page 30

Overall Study Results

- You are exposed to particles from traffic, cooking, and other sources
- We found fragrance chemicals – which are avoidable asthma triggers – in every home.

See page 8

We know more about the health effects of some chemicals than others. Clear connections between chemicals and health can't always be made.

What You Can Do

1. Avoid products marked as antibacterial.
2. Open windows or use an exhaust fan when cooking.
3. Fix rips in furniture so foam isn't exposed.
4. Choose products that are fragrance-free.

© 2015 Silent Spring Institute.
This work is licensed under a Creative Commons Attribution 4.0 International License.

Overall Study Results

What is the purpose of the study?

Many children in your community have asthma. Sometimes chemicals, mold, or dust can make asthma symptoms worse. Since some apartment buildings in your community are being renovated, we did this study to find out whether renovations are changing homes in any way that could increase or decrease breathing problems, including asthma.

You are exposed to particles from traffic, cooking, and other sources

Small particles in the air can travel deep inside the lung, and can affect your health. These particles, which may come from smoking, cooking or traffic, can trigger asthma attacks.

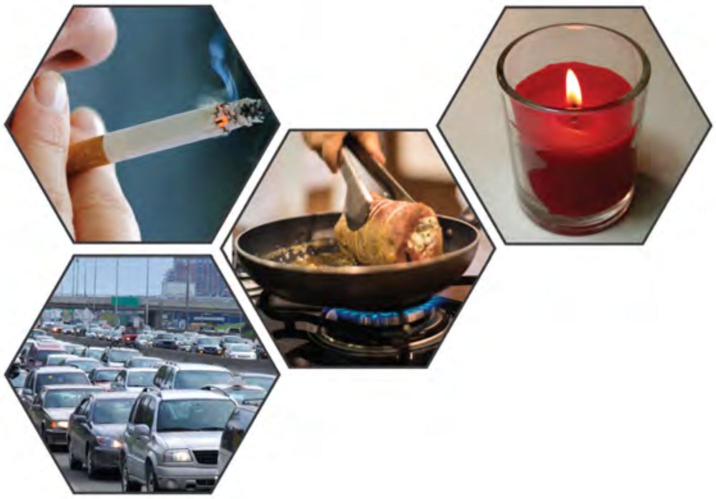

Tips for a healthy home

- do not smoke inside
- open windows or use exhaust fans when cooking foods that make a lot of smoke
- do not burn candles or incense when people with lung conditions are in the home.
- Ask your building manager to install "high-MERV" air filters in the building ventilation (heating/cooling) system to reduce particle pollution.

There are many asthma triggers in the home.

Some asthma triggers are related to pests, such as cockroaches and mice. We found mouse allergen in the dust in many homes, and many children were allergic to this allergen.

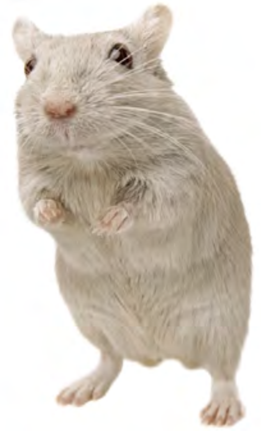

Tips for a healthy home

- keep pests out of your home by eliminating sources of food and water and sealing up holes and gaps
- limit the use of pesticides, which can be bad for your health

You are exposed to chemicals from the products you use.

You are exposed to chemicals in the products you use and the building materials around you. Exposure may be direct (for example, applying lotion) or indirect (for example, chemicals move out of your furniture and into your air and dust).

Tips for a healthy home

- Use only a few simple cleaners – water, fragrance-free soap, baking soda and vinegar – for many cleaning tasks.
- Open windows or use bathroom or kitchen vents when using products that have a strong smell.
- Keep dust levels low by wiping surfaces with a damp cloth and cleaning with a vacuum cleaner with a HEPA filter.

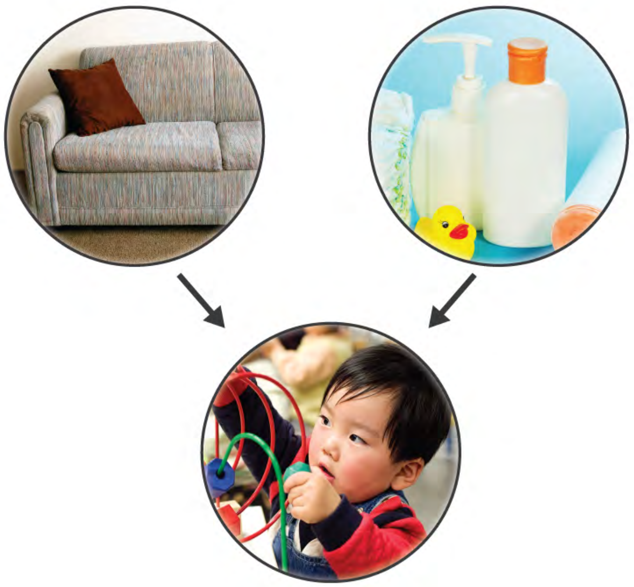

You may be directly exposed to chemicals in the products you buy and the food you eat. You could also be exposed to chemicals after they move out of your products into the dust or air in your home.

We found fragrance chemicals – which are avoidable asthma triggers – in every home.

Most of the homes also had antibacterial chemicals, another avoidable asthma trigger. These chemicals have been linked to worsening asthma symptoms.

Tips for a healthy home

- Choose fragrance-free and avoid anti-bacterial products.
- Soap and water are just as effective as antibacterial soap.

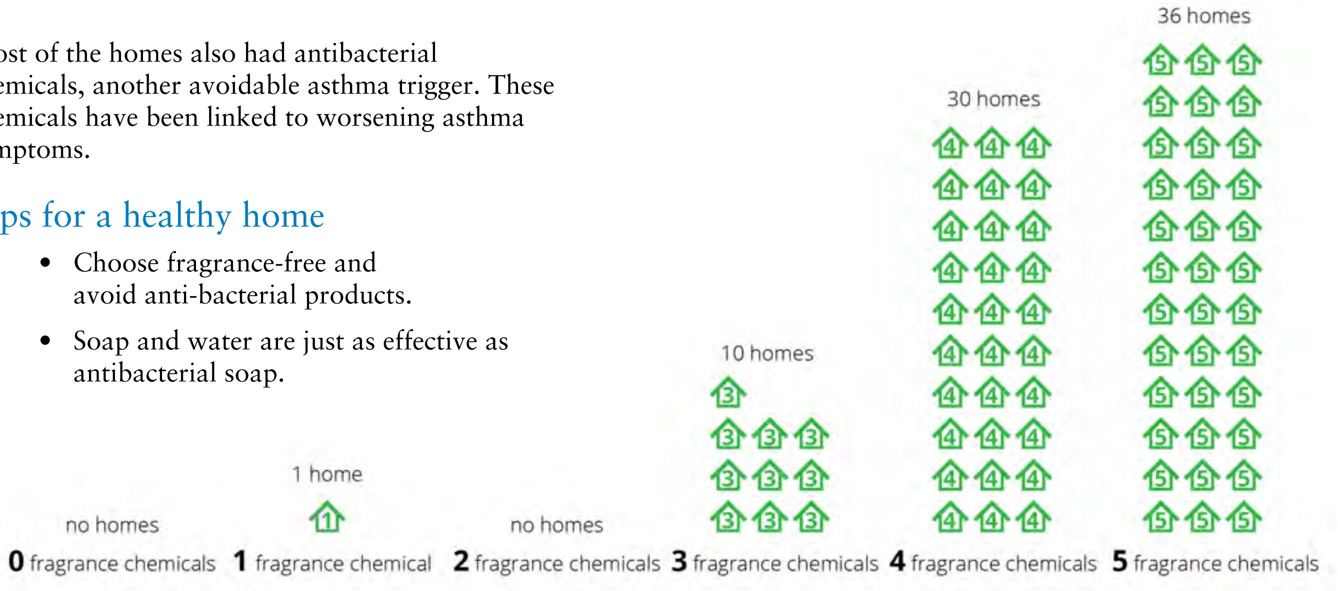

Number of fragrance chemicals in air

© 2015 Silent Spring Institute.
This work is licensed under a Creative Commons Attribution 4.0 International License.

How to Read Your Graphs

Later in this report, you will find information about the chemicals we tested and graphs of your results.

Each graph shows how much of a chemical we measured in your home or your child's urine sample. You might want to notice:

What kinds of chemicals were found?

Is my level for a chemical higher than others?

The graphs show the chemicals we found in your samples at levels that were higher than most of the others in the study. If your level of a chemical is a lot higher than others, that means you can probably make changes to lower the level.

If you would like help reading your graphs, please contact us by phone at 617-332-4288 or email at GHS@silentspring.org

Chemical group

Parabens

Chemical

Units of measure

Amount of chemical in urine, dust, or air. Higher numbers mean more chemical exposure.

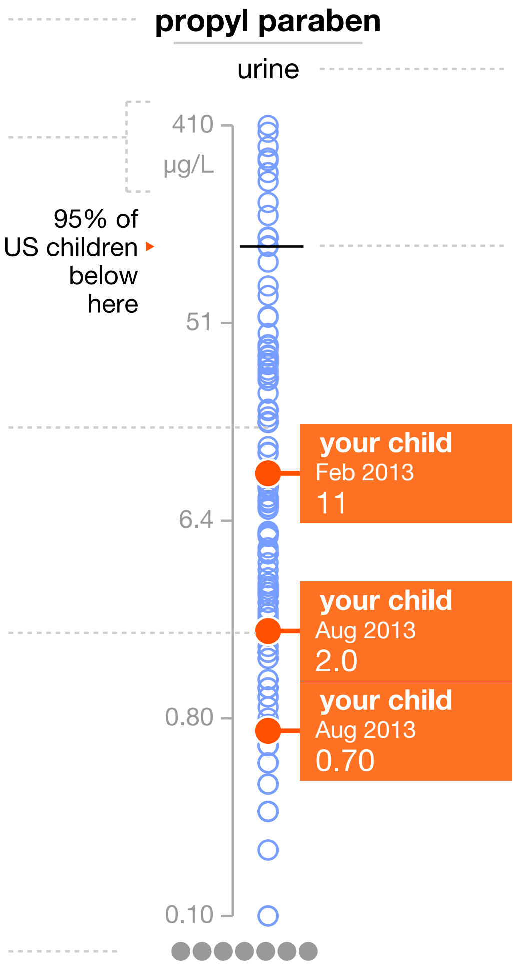

Tested in

We tested for chemicals in your air, dust, and child's urine

Percentiles

In a national survey of children, 95% had levels below this point.

Other children in the study

Each blue circle is another child's chemical level

Your child's chemical levels

The orange circles are your child's chemical levels

Not detected

The chemical was below the limit of detection in these children's urine. The chemical might not be there or it might be a very low level that we couldn't measure.

 © 2015 Silent Spring Institute.
This work is licensed under a Creative Commons Attribution 4.0 International License.

Asthma

We found 5 asthma related chemicals in your samples. Most people had less than 13.5.

How chemicals relate to asthma

A main focus of this study was on asthma. We are studying these chemicals because we suspect they might be connected with asthma symptoms.

General tips for a healthy home

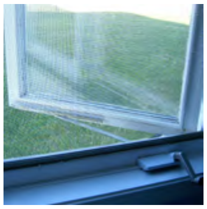

In your home

- **Burning anything in your house, whether smoking or cooking, can release chemicals that can trigger asthma symptoms.** Don't allow anyone to smoke in your home. Open the window or use an exhaust fan when cooking.
- **Fragrances can irritate lungs.** Many products contain fragrances, including air fresheners, laundry detergents, diapers, and soaps. You can buy products that are fragrance free.
- **Try to reduce the use of chemical cleaners and pesticides in your home.** Some household cleaners can irritate eyes or lungs.
- **Wiping with a damp cloth or mop captures dust and prevents chemicals and allergens in the dust from getting into the air.** So mop rather than sweep, wet-wipe surfaces to remove dust, or use a vacuum with a HEPAi (high-efficiency particulate absorption) filter if you can.

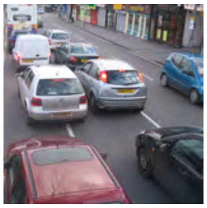

Community Actions

- **Idling cars can put particulate matter in your air.** Anti-idling ordinances have improved air quality in some communities. Try to avoid idling your car and encourage your neighbors to do the same.
- **Talk to your building manager about using integrated pest management (IPM) to decrease the use of pesticides in your building.** You can help by eliminating open food sources, including food for pets. Also, check for and repair drips or leaks, and reduce clutter in your home. Share the information you've learned with your neighbors.

© 2015 Silent Spring Institute.
This work is licensed under a Creative Commons Attribution 4.0 International License.

Known links to asthma

Children show asthma symptoms when they're exposed to things they're allergic to. Also, some chemicals irritate the respiratory system and can cause asthma symptoms.

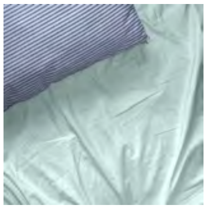

Dust mite allergen

House dust mites are tiny bugs that you cannot see. They live in fabric and can be found in mattresses, pillows, cloth-covered furniture, carpeting, and stuffed toys.

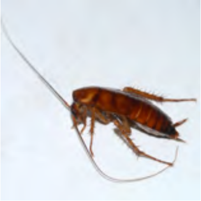

Cockroach allergen

Some children are sensitive to the allergens that cockroaches leave behind. Pests need the same things humans do: food, water, shelter, and a way to come and go. Cockroaches like kitchens because they are warm and provide sources of food and water.

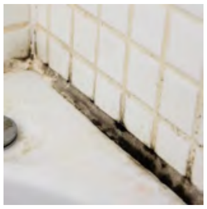

Mold-related chemical

Too much water in your home or humidity in the air can lead to mold growth or pest problems. For example, after a water leak, mold can grow on walls and floors. This mold can lead to breathing problems, especially for sensitive individuals.

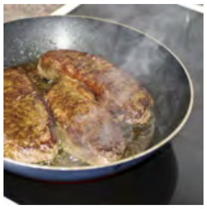

Particulate Matter

Particles in the air from traffic, cooking, and smoking can be inhaled and trigger asthma attacks.

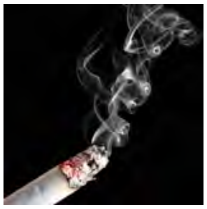

Tobacco smoke exposure

First and secondhand smoke comes from a burning cigarette, cigar, or pipe, or from an exhaling smoker. Second hand smoke exposure has been show to trigger asthma attacks in young children.

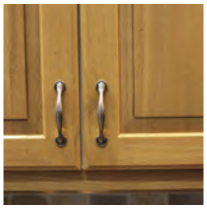

Formaldehyde

Formaldehyde is found in furniture made from particle board and in insulation, carpet, and cigarette smoke. Formaldehyde levels go up with higher temperature and humidity.

Possible links to asthma

Other chemicals we tested for may also affect asthma and the respiratory system. We don't yet have enough evidence to be sure, but you might still consider taking steps to reduce your exposure to these chemicals.

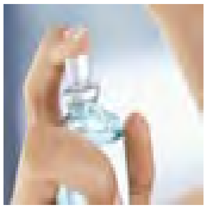

Fragrance Chemicals

We don't usually think of fragrances as chemicals, but they are. Fragrance chemicals are used to create artificial smells or mask the odor of other ingredients. Fragrances are found in many commonly used products such as soap, shampoo, deodorant, lotion, powder, candles, air freshener, and laundry and cleaning products.

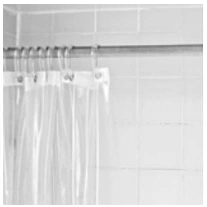

Phthalates (pronounced tha-lates)

Phthalates are chemicals used to make plastics more flexible and to keep the color and scent in personal care products. They are found in many products such as vinyl shower curtains, nail polish, food containers, toys, and plastic bags.

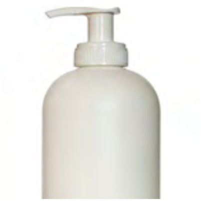

Antibacterials

Antibacterials kill bacteria and are sometimes found in household cleaning products, soaps, and hand sanitizers. One reason to avoid anti-bacterial products is the active ingredient triclosan. The American Medical Association recommends that triclosan not be used in the home, as it may boost bacterial resistance to antibiotics.

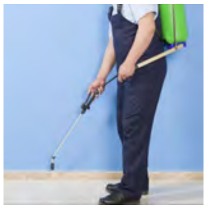

Pesticides

Pesticides are chemicals that kill insects, ants, termites, mold, weeds, and other pests.

We also measured chemicals that might change when a building is renovated. These chemicals are not expected to affect asthma, but they may affect health in other ways. Your results for these chemicals are included, too.

© 2015 Silent Spring Institute.
This work is licensed under a Creative Commons Attribution 4.0 International License.

Your Results:

Asthma

FRAGRANCE CHEMICALS	PHTHALATES	TOBACCO	ANTIBACTERIALS
Chemicals used to create artificial smells or mask unwanted odors	Found in some plastics and personal care products	Chemicals found in tobacco or breakdown products of nicotine	Chemicals that kill bacteria found in soaps and hand sanitizers
You were below the median in all fragrance chemicals.	You were below the median in all phthalates.	You were below the median in all of these chemicals.	You were below the median in all antibacterials.

your chemical level

other people's chemical levels

households where the chemical was not detected

 © 2015 Silent Spring Institute.
This work is licensed under a Creative Commons Attribution 4.0 International License.

Pesticides

Chemicals that kill insects, ants, termites, mold, weeds, and other pests

You were below the median in most **pesticides.**

Turn the page to see your results.

We found 3 pesticides in your samples. Most people had less than 6.

Our methods could detect very low levels of pesticides that may have been too low to be detected by other tests.

How pesticides get in your home

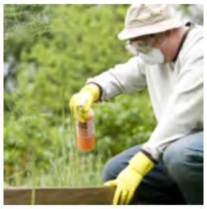

- Pesticides can get into your home in bug sprays, cleaners, or mothballs.
- Pesticides from outside might come into your home on your shoes or pets.
- Children can be exposed to pesticides outside the home, too.
- Some of the pesticides we tested for have been banned. They are still found in homes, because they can stay on indoor surfaces and in dust for a long time.

Pesticides and health

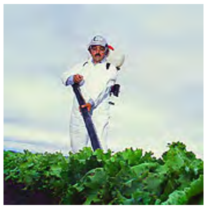

- Pesticides can cause many types of health effects. They can affect the brain, reproductive system, hormones, and how well the body fights off disease.
- The Environmental Protection Agency reviews the safety of pesticides. They phased out use of chlorpyrifos (in 2000) and diazinon (in 2004) in home products because of health concerns.

What you can do for a healthy home

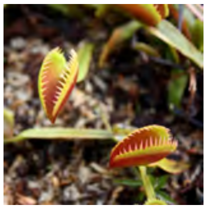

Consider alternatives for controlling pests

- **Be less appetizing:** Keep food in sealed containers.
- **Be less inviting:** Seal cracks and crevices to keep pests out.
- **Kill without chemicals:** Use traps instead of sprays.

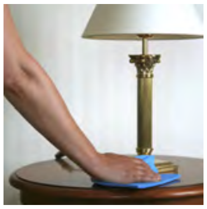

Keep pesticides levels low

- **Pesticides may be in your house dust.** Try to keep dust levels low. Wipe floors and surfaces with a damp cloth or mop. Or use a vacuum with a HEPA (high-efficiency particulate air) filter to prevent dust from getting back into the air.
- **Keep pesticides from getting in.** Place rugs in doorways to avoid tracking pesticides into your home. Take off your outdoor shoes at the door.

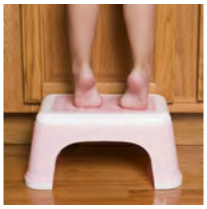

If you must use pesticides...

- Store pesticides out of the reach of children.

© 2015 Silent Spring Institute.
This work is licensed under a Creative Commons Attribution 4.0 International License.

Your Results:

Pesticides

WEED KILLERS

Chemicals used to control weeds, also found in disinfectants

You were below the median in all weed killers.

OTHER PEST CONTROL CHEMICALS

Lice and flea control, mothballs

You were below the median in all of these chemicals.

ORGANOPHOSPHATES

Nerve agents used to control insects

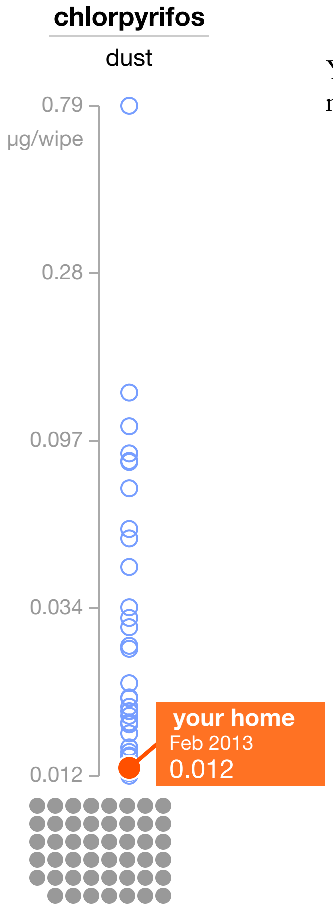

You were below the median in

- diazinon
- propoxur

your chemical level | other people's chemical levels | households where the chemical was not detected

Your Results:

Pesticides

PYRETHROIDS	BANNED ORGANOCHLORINES	INSECT REPELLANTS
Common chemicals used to control insects	Chemicals used to control insects	
You were below the median in all pyrethroids.	You were below the median in all banned organochlorines.	

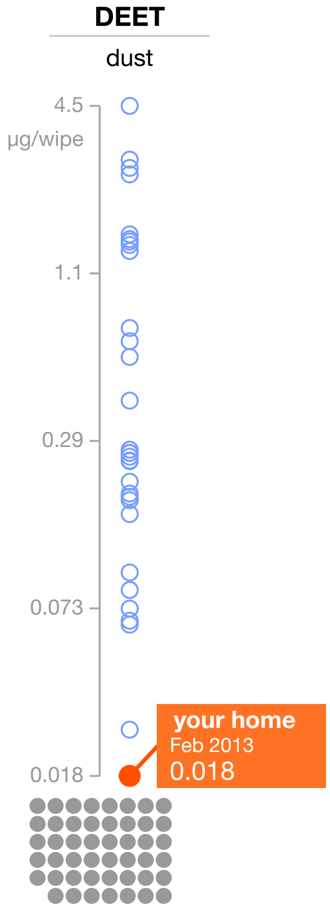

your chemical level

other people's chemical levels

households where the chemical was not detected

SILENT SPRING INSTITUTE silentspring.org © 2015 Silent Spring Institute.
This work is licensed under a Creative Commons Attribution 4.0 International License.

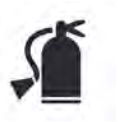

Flame Retardants

Chemicals added to many products to make it harder for them to catch fire.

Your samples had a higher level of a **flame retardant** than most others in the study.

Turn the page to see your results.

We found 6 flame retardants in your samples. Most people had less than 11.

How flame retardants get in your home

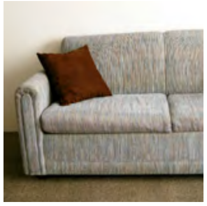

- Flame retardants are added to products such as furniture foam, electronics, and cloth.
- These chemicals come out of products and collect in house dust. People accidentally eat dust when it gets on food or hands. Babies and toddlers are more exposed to dust, because they spend time on the floor and put things in their mouths.

Flame retardants and health

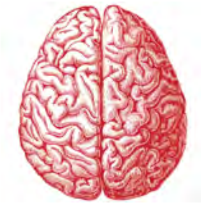

- Some flame retardants might cause cancer. Others affect thyroid hormones or the brain and nervous system, which means that they can affect brain development and IQ, weight, depression, energy, and muscle control. Some flame retardants haven't been studied yet for health effects.
- Some flame retardants are banned in Europe and in many states because they affect thyroid hormones.

What you can do for a healthy home

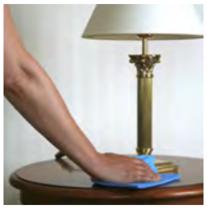

Dust

- **Flame retardants can stick around your house in dust.** Try to keep dust levels low. For example, wipe surfaces with a damp cloth or mop. Or use a vacuum with a HEPA (high-efficiency particulate air) filter to prevent dust from getting back into the air.
- **Wash your hands often.**

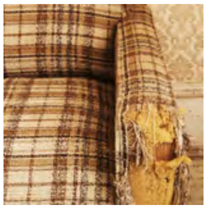

Furniture

- **Fix rips in furniture** so foam isn't exposed.
- **When you buy furniture, ask for furniture that doesn't contain flame retardants, including in the foam.** Or choose furniture made from wool, hemp, polyester, latex, down, or leather, because these materials are naturally slow to catch fire. Choose rug pads made from felt, jute, or rubber instead of foam.

© 2015 Silent Spring Institute.
This work is licensed under a Creative Commons Attribution 4.0 International License.

Your Results:

Flame Retardants

BROMINATED FLAME RETARDANTS

Flame retardants in furniture foams and electronics. Most of these chemicals are being phased out of use

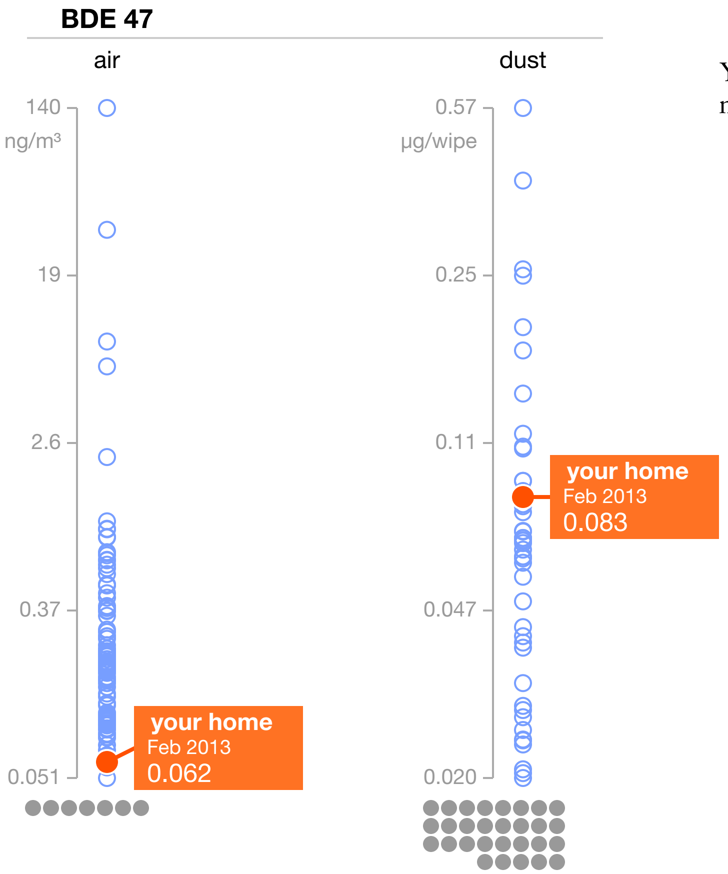

You were below the median in

- 22BBM13P
- 23DB1P

your chemical level

other people's chemical levels

households where the chemical was not detected

Your Results:
Flame Retardants

FIREMASTER 550

Replacement for brominated flame retardants in furniture foams

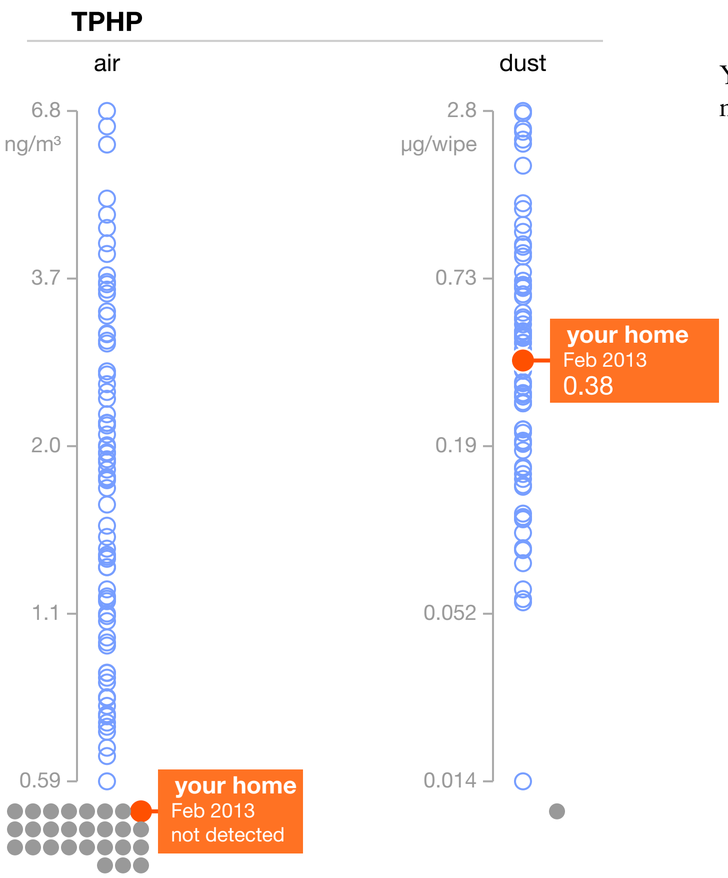

You were below the median in
- BEH-TEBP
- EH-TBB

your chemical level | other people's chemical levels | households where the chemical was not detected

SILENT SPRING INSTITUTE silentspring.org © 2015 Silent Spring Institute.
This work is licensed under a Creative Commons Attribution 4.0 International License.

Your Results:

Flame Retardants

NON-HALOGENATED ORGANOPHOSPHATES

Often used in plastics and flame retardant mixtures

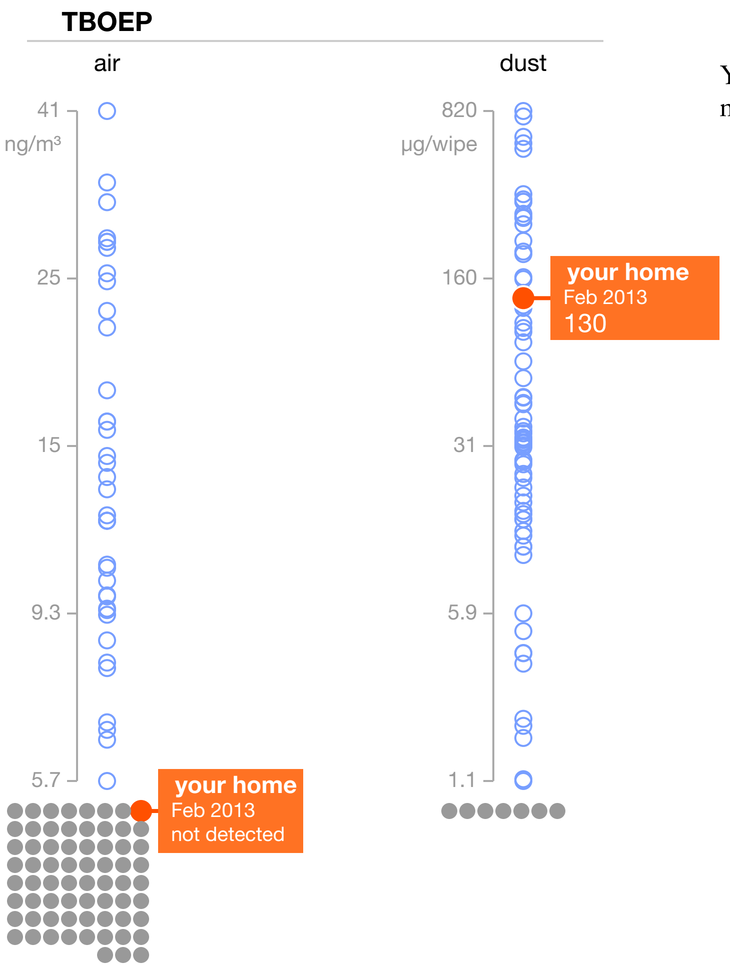

You were below the median in

- TBOPP
- TBPP
- TMPP

your chemical level

other people's chemical levels

households where the chemical was not detected

Your Results:

Flame Retardants

HALOGENATED ORGANOPHOSPHATES

Widely used flame retardants found in furniture foams, plastics, textiles, and insulation

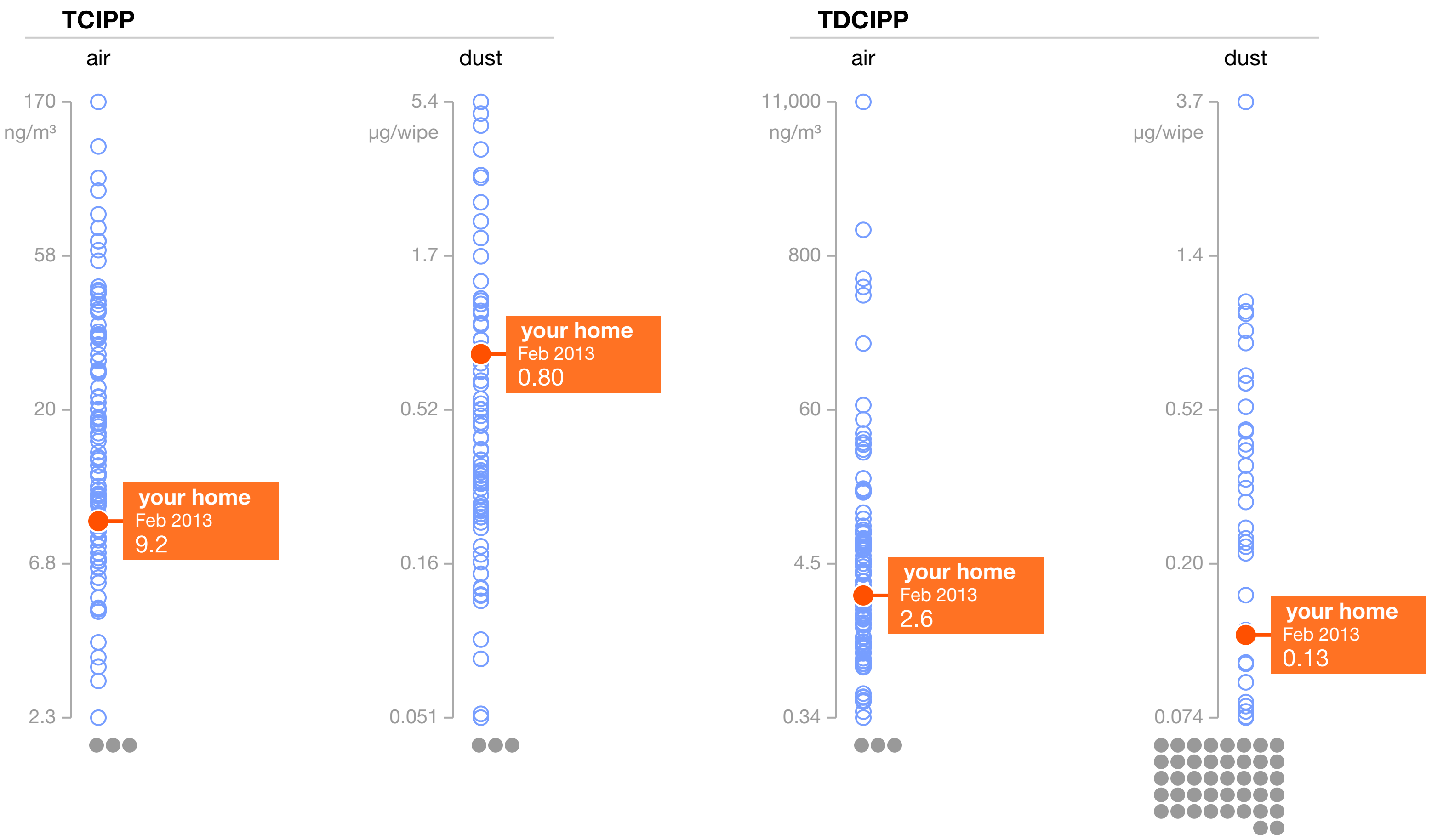

your chemical level

other people's chemical levels

households where the chemical was not detected

SILENT SPRING INSTITUTE silentspring.org © 2015 Silent Spring Institute.
This work is licensed under a Creative Commons Attribution 4.0 International License.

Your Results:

Flame Retardants

HALOGENATED ORGANOPHOSPHATES

Widely used flame retardants found in furniture foams, plastics, textiles, and insulation

You were below the median in

- TCEP
- TDCIPP related compound

your chemical level | other people's chemical levels | households where the chemical was not detected

© 2015 Silent Spring Institute.
This work is licensed under a Creative Commons Attribution 4.0 International License.

Fragrances

Your samples had lower levels than most people for **fragrance chemicals.**

Turn the page to see your results.

We found 3 fragrance chemicals in your samples. Most people had less than 8.5.

How fragrances get in your home

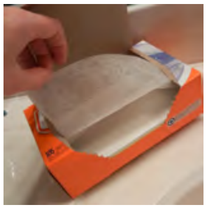

- Fragrance chemicals are sometimes in products such as soap, shampoo, and cosmetics. They are sometimes in cleaners, laundry products, dryer sheets, diapers, and air fresheners. Dryer sheets and air fresheners have a lot of fragrance.

Fragrances and health

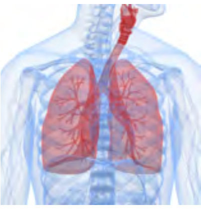

- Some fragrance chemicals may increase asthma symptoms.
- Some fragrance chemicals are weak estrogen mimics, so they may affect breast development, puberty, fertility, and some cancers. Scientists are working to learn more.

What you can do for a healthy home

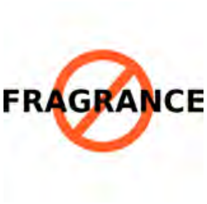

Avoid products with fragrances

- Don’t use air fresheners or dryer sheets.
- Use products that are fragrance-free.

SILENT SPRING INSTITUTE silentspring.org © 2015 Silent Spring Institute.
This work is licensed under a Creative Commons Attribution 4.0 International License.

Your Results:
Fragrances

FRAGRANCE CHEMICALS

Chemicals used to create artificial smells or mask unwanted odors.

You were below the median in all fragrance chemicals.

your chemical level

other people's chemical levels

households where the chemical was not detected

 © 2015 Silent Spring Institute.
This work is licensed under a Creative Commons Attribution 4.0 International License.

Combustion Byproducts - PAHs

Formed when things such as gasoline and diesel are burned

Your samples had higher levels of **combustion byproducts** than most others in the study.

Turn the page to see your results.

We found 4 combustion byproducts in your samples. Most people had less than 3.5.

How PAHs get in your home

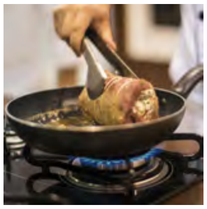

- Combustion byproducts known as PAHs (polycyclic aromatic hydrocarbons) can be found in gasoline, vehicle exhaust, tobacco smoke, wood fires, cooking fumes, and grilled food.

PAHs and health

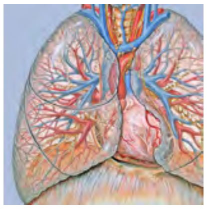

- These chemicals can cause breathing problems, heart disease as well as lung and other cancers.

What you can do for a healthy home

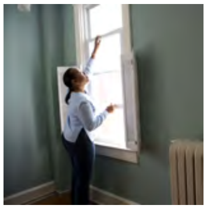

Your home

- **Tobacco smoke is a source of PAHs.** Don't smoke and don't allow smoking in your home.
- **Chemicals in the air can build up indoors.** Turn on the exhaust fan or open the window when you are cooking. Vent your gas stove, broiler, grill, or fireplace to the outdoors.

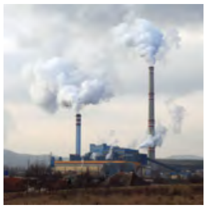

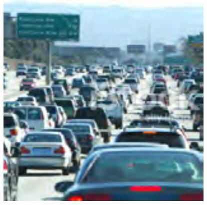

Your community

- **Some of your home's air quality comes from your community's air.** Work towards improving the air in your whole neighborhood by advocating for air pollution controls and anti-idling rules. Work to support public transportation and switching buses and trucks away from diesel to safer fuels.
- **Ask your building manager to install "high-MERV" air filters** in the building ventilation (heating/cooling) system to reduce particle pollution.
- **Work with your building manager to reduce smoking,** enforce no-smoking rules, and help residents quit.

© 2015 Silent Spring Institute.
This work is licensed under a Creative Commons Attribution 4.0 International License.

Your Results:

Combustion Byproducts - PAHs

PAHS

Combustion byproducts that can be found in gasoline, vehicle exhaust, tobacco smoke, wood fires, cooking fumes, and grilled food

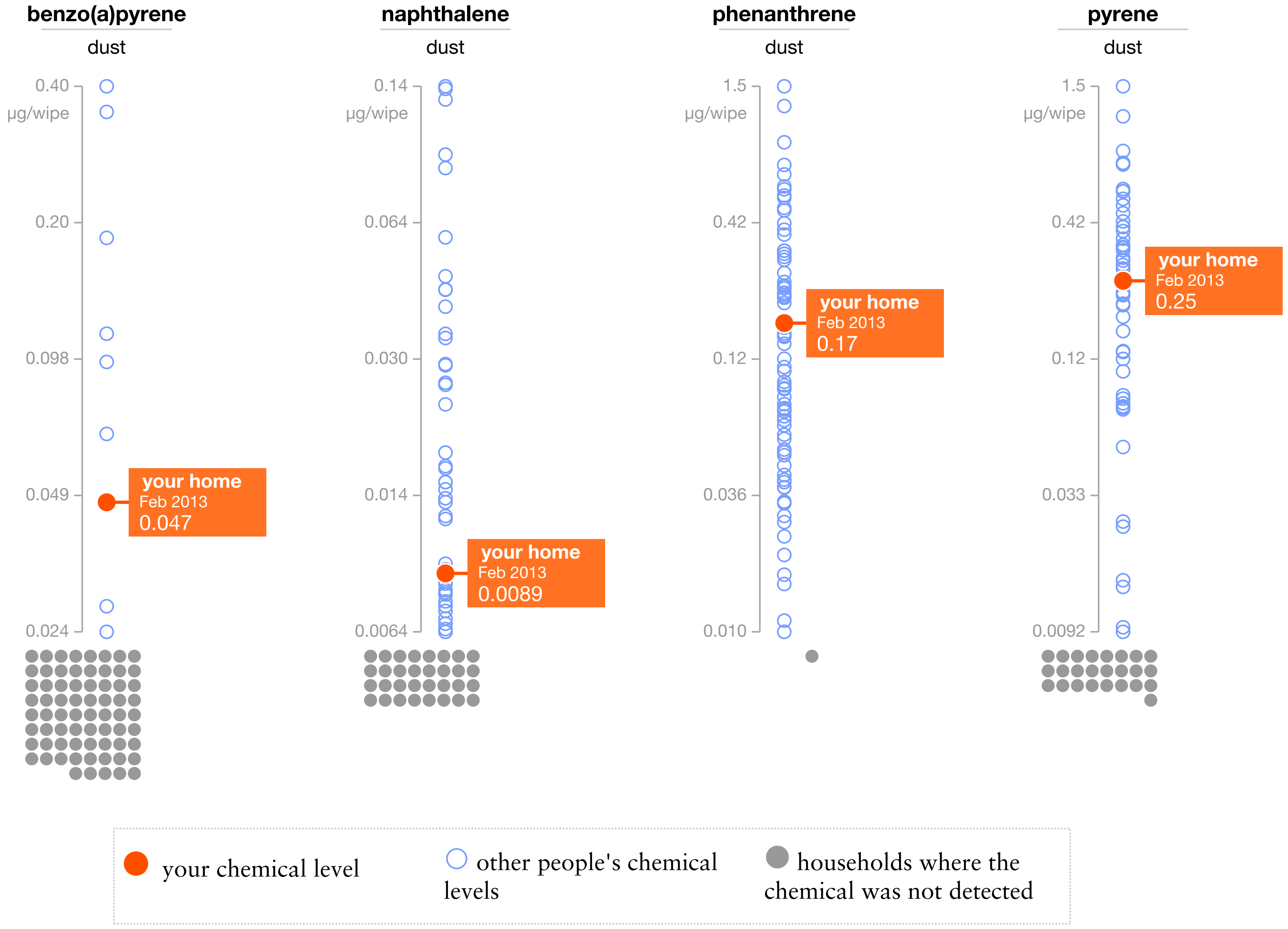

Your Results:

Combustion Byproducts - PAHs

TOBACCO

Chemicals found in tobacco or breakdown products of nicotine

You were below the median in all of these chemicals.

your chemical level

other people's chemical levels

households where the chemical was not detected

SILENT SPRING INSTITUTE silentspring.org © 2015 Silent Spring Institute.
This work is licensed under a Creative Commons Attribution 4.0 International License.

Banned Industrial Chemicals

Used in electronic equipment and plastics until 1979

Your samples had lower levels than most people for **banned industrial chemicals.**

Turn the page to see your results.

We found 0 banned industrial chemicals in your samples. Most people had 0.

How PCBs get in your home

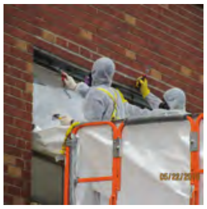

- PCBs were used in electronic equipment, floor finishes, and construction materials before they were banned in the U.S. in 1979.
- PCBs are found in pre-1979 fluorescent light ballasts, caulk, and the air, dust, and soil in and around older buildings.
- They are also found in fish from polluted waters and soil in former industrial areas.
- PCB 11 is an unintentional byproduct found in commercial pigments used in paints, inks, and other materials in the home. Pigments made in the U.S. are less likely to contain these PCB contaminants

PCBs and health

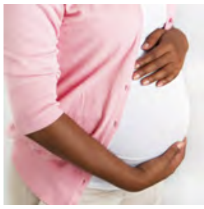

- PCBs affect thyroid hormones. Exposure during pregnancy affects the baby's brain development and IQ. They may increase breast cancer risk in some people.
- EPA banned the use of PCBs in 1979 because of health concerns.

What you can do for a healthy home

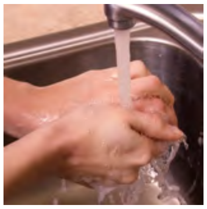

Dust

- **PCBs, although banned, can still be in dust in older homes.** Keep dust levels low. Wipe floors and surfaces with a damp cloth or mop. Or use a vacuum with a HEPA (high-efficiency particulate air) filter to prevent dust from getting back into the air.
- **Wash hands often.**

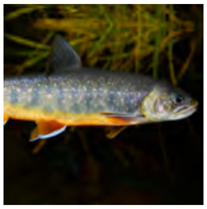

Food

- **If you are fishing, observe signs about eating the fish.** In the grocery store, bluefish, swordfish, and shark can have high levels.
- **PCBs accumulate in fat.** Trim off skin and fat from fish and meat and let fat drain off before cooking. Choose low-fat dairy.

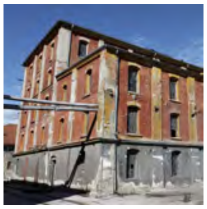

Buildings

- **Buildings constructed before 1979 may have used contaminated materials.** Avoid touching caulk or surfaces near caulk in pre-1979 buildings.
- **Be careful when renovating older buildings.** Follow EPA guidelines for managing PCB-containing materials.
- **Some paints made outside of the US can contain PCB 11.** Look for products made in the US.

 © 2015 Silent Spring Institute.
This work is licensed under a Creative Commons Attribution 4.0 International License.

Your Results:

Banned Industrial Chemicals

PCBS

Industrial chemicals banned in 1979

You were below the median in all PCBs.

your chemical level

other people's chemical levels

households where the chemical was not detected

 © 2015 Silent Spring Institute.
This work is licensed under a Creative Commons Attribution 4.0 International License.

Personal Care Products

Chemicals found in hair and body care products and cosmetics

Your home had one of the highest levels of a **personal care product.**

Turn the page to see your results.

We found 8 personal care products in your samples. Most people had less than 13.

How these chemicals get in your home

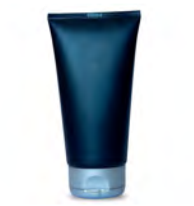

- Chemicals in products such as soap, shampoo, deodorant, lotion, hair products, cosmetics, and sunscreen.
- Chemicals come out of these products into the air and dust.

Personal care product chemicals and health

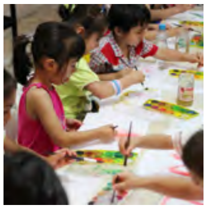

- Many of these chemicals are hormone disruptors. Some mimic estrogen or block androgen, so they may affect child development, fertility, and some cancers. Scientists are working to learn more.

What you can do for a healthy home

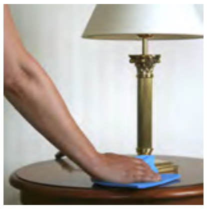

Dust

- **Keep dust levels low.** Wipe surfaces with a damp cloth and use a vacuum with a HEPA (high-efficiency particulate air) filter to prevent dust from getting back into the air.

Products

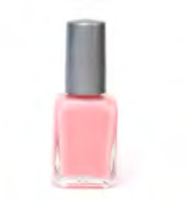

- **Avoid using nail polish or apply it outdoors.**
- **Use products that are fragrance-free.**

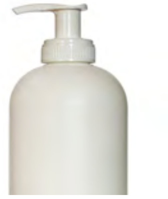

- **Avoid products marked as antibacterial.** These products may contain triclosan, a chemical we tested for in this study.
- **Learn which companies avoid harmful chemicals.** Look for labels that say “phthalate-free” and ask stores and manufacturers whether products are phthalate-free.

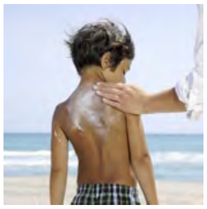

- **Avoid products with parabens listed on the label.** Common names for these chemicals include butyl paraben, ethyl paraben, methyl paraben, and propyl paraben.
- **Sunscreens may contain chemicals of concern.** Choose shade, hats, and tightly woven clothing instead of sun screen when you can.

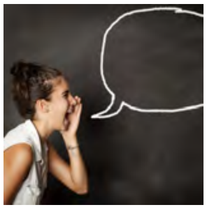

Speak out

- **Ask your favorite brands and stores to choose safer chemicals** and join campaigns to get chemicals of concern out of consumer products.

© 2015 Silent Spring Institute.
This work is licensed under a Creative Commons Attribution 4.0 International License.

Your Results:

Personal Care Products

SUNSCREEN CHEMICALS

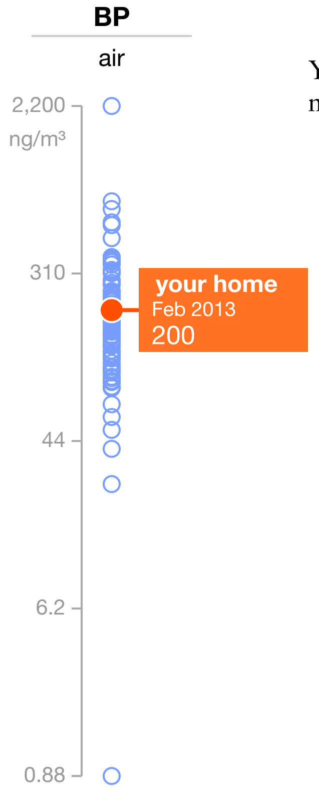

You were below the median in

- BP-3

PARABENS

Preservatives that can be found in shampoos, toothpaste, gels, and other personal care products

You were below the median in all parabens.

your chemical level

other people's chemical levels

households where the chemical was not detected

Your Results:
Personal Care Products

PHTHALATES

Additives found in some personal care products

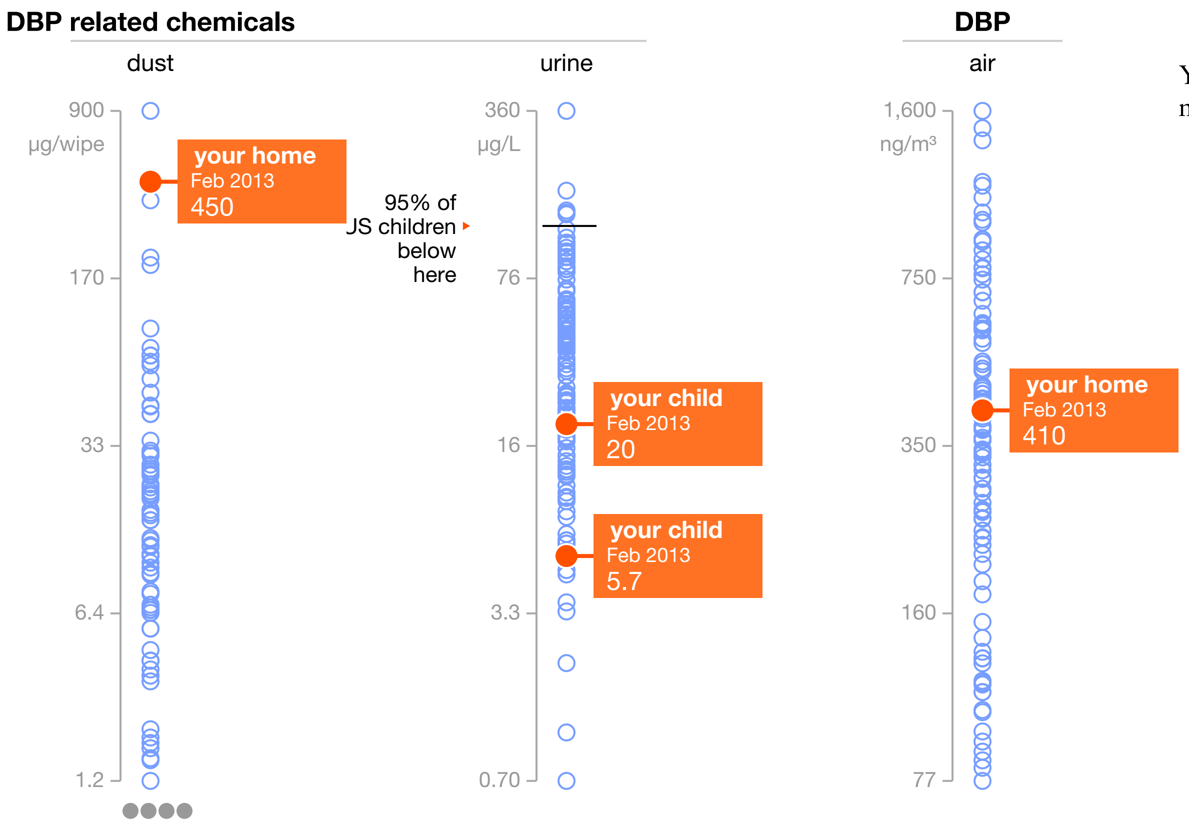

You were below the median in

- mCPP

your chemical level | other people's chemical levels | households where the chemical was not detected

SILENT SPRING INSTITUTE silentspring.org © 2015 Silent Spring Institute.
This work is licensed under a Creative Commons Attribution 4.0 International License.

Your Results:

Personal Care Products

ANTIBACTERIALS

Chemicals that kill bacteria found in soaps and hand sanitizers

You were below the median in all antibacterials.

your chemical level

other people's chemical levels

households where the chemical was not detected

 © 2015 Silent Spring Institute.
This work is licensed under a Creative Commons Attribution 4.0 International License.

Plastics Chemicals

Chemicals that are added to plastics

You were below the median in most **plastics chemicals**.

Turn the page to see your results.

We found 9 plastics chemicals in your samples. Most people had less than 17.

How plastics chemicals get in your home

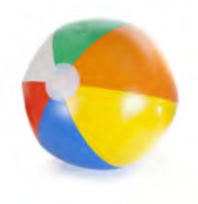

- These chemicals are found in vinyl products, food packaging, plastic toys, furniture, and other products.
- They come out of these products and collect in household dust.

Plastics chemicals and health

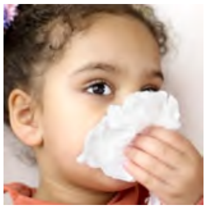

- Many of these chemicals are hormone disruptors. Some mimic estrogen or block androgen, so they may affect child development, fertility, and some cancers. Scientists are working to learn more.
- Some of these chemicals contribute to allergies and asthma.
- Some phthalates were banned in kids toys and cosmetics in Europe because of concerns about their effects on children's development.

What you can do for a healthy home

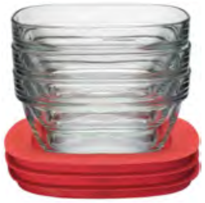

Your food

- **Chemicals may come out of plastic containers into your food or drinks.** Use stainless steel or glass containers for food and drinks. Avoid microwaving food in plastic containers. Brew coffee in a French press instead of a plastic coffee maker.
- **Processed foods tend to have more of these chemicals than fresh foods.** Cook more meals at home with fresh ingredients.
- **Chemicals used in food cans may get into your food.** Choose fresh or frozen instead of canned food or drinks.

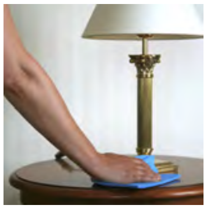

Your home

- **Find alternatives to plastic.** Go plastic- and vinyl-free when possible, including for shower curtains (choose untreated cloth or nylon instead), toys and flooring.
- **Chemicals in plastic end up in your dust.** Keep dust levels low. For example, wipe surfaces with a damp cloth and use a vacuum with a HEPA (high-efficiency particulate air) filter to keep dust from getting back into the air.

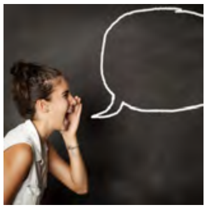

Your life

- **Advocate for a change in how consumer products and food packaging are made.** Ask your favorite brands and stores to choose safer chemicals and join campaigns to get chemicals of concern out of food packaging and consumer products.

 © 2015 Silent Spring Institute.
This work is licensed under a Creative Commons Attribution 4.0 International License.

Your Results:

Plastics Chemicals

PHTHALATES

Plasticizers found in some plastics including vinyl plastics

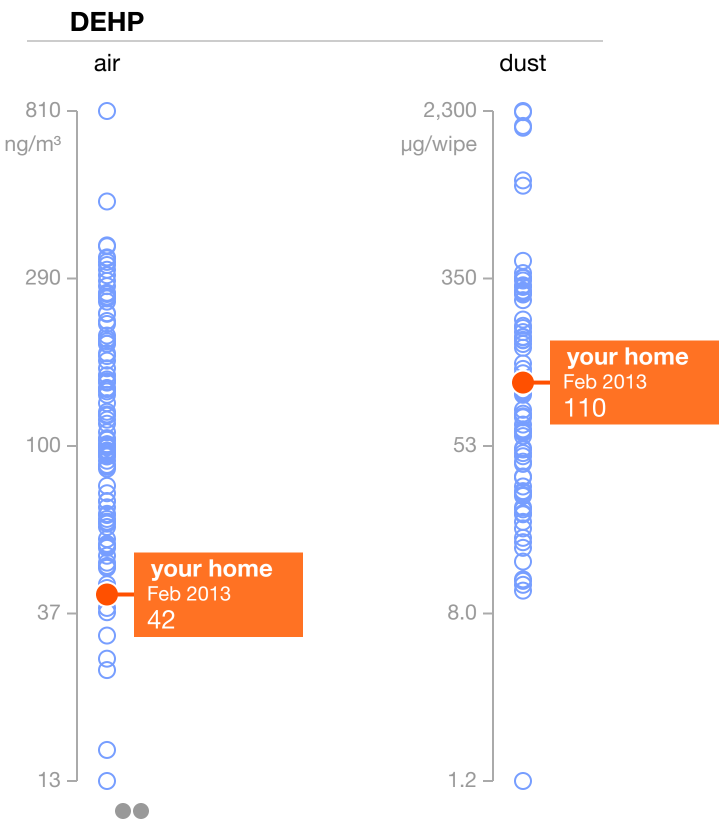

your chemical level

other people's chemical levels

households where the chemical was not detected

Your Results:
Plastics Chemicals

PHTHALATES

Plasticizers found in some plastics including vinyl plastics

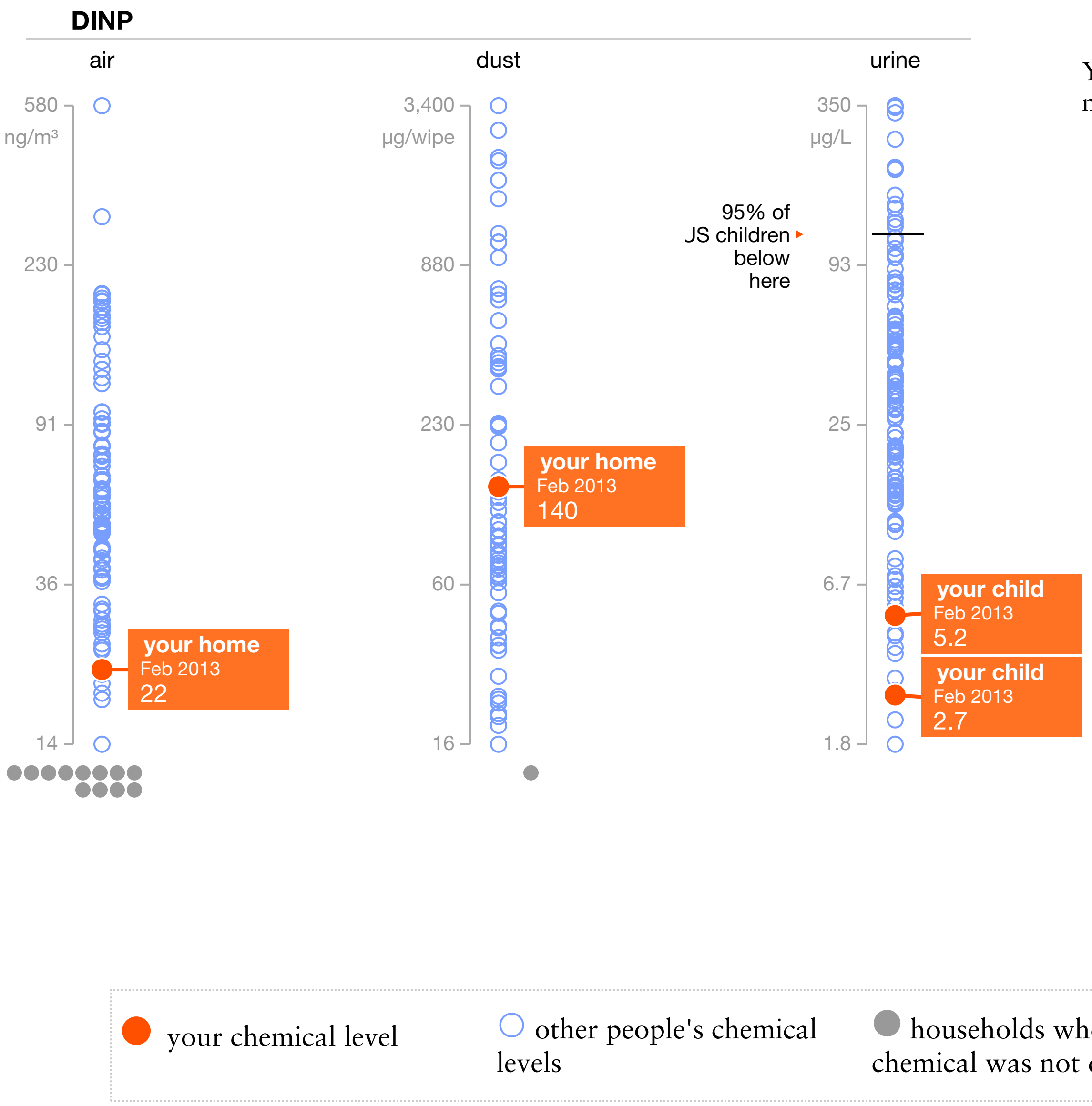

You were below the median in

- BBP
- DCHP
- DEHP related chemicals
- mCNP
- MiBP

SILENT SPRING INSTITUTE silentspring.org © 2015 Silent Spring Institute.
This work is licensed under a Creative Commons Attribution 4.0 International License.

Your Results:

Plastics Chemicals

BISPHENOLS

Used in consumer plastics as plasticizers, especially for clear plastic products such as water bottles

You were below the median in all bisphenols.

PLASTICIZERS

Used to increase plasticity

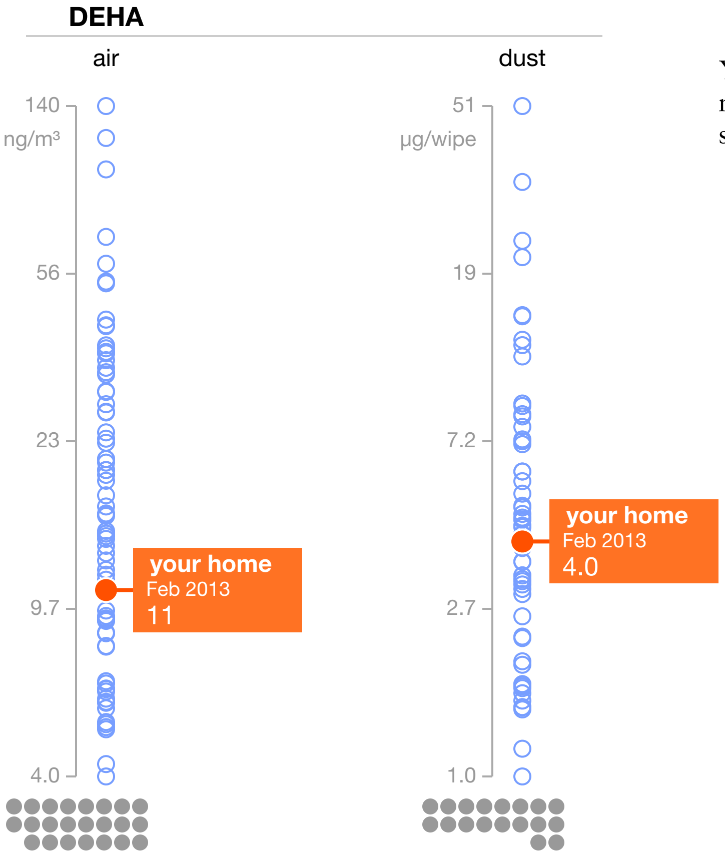

PHTHALATE SUBSTITUTES

Additives found in some plastics used instead of phthalates

You were below the median in all phthalate substitutes.

- your chemical level
- other people's chemical levels
- households where the chemical was not detected

Your Results:

Plastics Chemicals

ALKYLPHENOLS

Plasticizer

You were below the median in all alkylphenols.

your chemical level

other people's chemical levels

households where the chemical was not detected

SILENT SPRING INSTITUTE silentspring.org © 2015 Silent Spring Institute.
This work is licensed under a Creative Commons Attribution 4.0 International License.

Appendix

The appendix lists every chemical in this report with how much of it was found in your home or your child's urine. If a chemical was "not detected", that means the chemical was below the limit of detection. The chemical might not be there at all, or it might be at a very low level that we couldn't measure.

CHEMICAL	FULL NAME	VISIT	DUST (µg/wipe)	AIR (ng/m³)	URINE (µg/L)	CATEGORY
22BBM13P	2,2-bisbromomethyl-1,3-propanediol	Feb 2013	—	not detected	—	Flame Retardants
23DB1P	2,3-dibromo-1-propanol	Feb 2013	—	not detected	—	Flame Retardants
24-DCP	2 4-dichlorophenol	Feb 2013	—	—	not detected	Pesticides
25-DCP	2 5-dichlorophenol	Feb 2013	—	—	not detected	Pesticides
AHTN	6-acetyl-1,1,2,4,4,7- hexamethyltetraline	Feb 2013	0.038	19	—	Asthma, Fragrance Chemicals
BBP	butylbenzyl phthalate	Feb 2013	290	42	—	Asthma, Plastics Chemicals
	monobenzyl phthalate	Feb 2013	—	—	5.2	Asthma, Plastics Chemicals
BDE 47	2,2′,4,4′-tetra- bromodiphenyl ether	Feb 2013	0.083	0.062	—	Flame Retardants
BEH-TEBP	bis(2-ethylhexyl)tetrabromophthalate	Feb 2013	—	not detected	—	Flame Retardants

CHEMICAL	FULL NAME	VISIT	DUST (µg/wipe)	AIR (ng/m³)	URINE (µg/L)	CATEGORY
benzo(a)pyrene		Feb 2013	0.047	—	—	Combustion Byproducts - PAHs
BP	benzophenone	Feb 2013	—	200	—	Personal Care Product Chemicals
BP-3	benzophenone-3	Feb 2013	—	11	1.6	Personal Care Product Chemicals
BPA	bisphenol A	Feb 2013	—	—	0.40	Plastics Chemicals
butyl paraben		Feb 2013	—	not detected	not detected	Personal Care Product Chemicals
carbaryl		Feb 2013	not detected	—	—	Pesticides
chlorpyrifos		Feb 2013	0.012	—	—	Pesticides
cotinine		Feb 2013	not detected	—	—	Asthma, Combustion Byproducts - PAHs
cypermethrin		Feb 2013	not detected	—	—	Pesticides
DBP	di-n-butyl phthalate	Feb 2013	—	410	—	Personal Care Product Chemicals
DBP related chemicals		Feb 2013	450	—	—	Personal Care Product Chemicals

SILENT SPRING INSTITUTE silentspring.org © 2015 Silent Spring Institute.
This work is licensed under a Creative Commons Attribution 4.0 International License.

Appendix

CHEMICAL	FULL NAME	VISIT	DUST (µg/wipe)	AIR (ng/m³)	URINE (µg/L)	CATEGORY
	mono-n-butyl phthalate	Feb 2013	—	—	5.7	Personal Care Product Chemicals
DCHP	di-cyclohexyl phthalate	Feb 2013	not detected	not detected	—	Plastics Chemicals
DDT	4,4'-DDT	Feb 2013	not detected	—	—	Pesticides
DEET	diethyltoluamide	Feb 2013	0.018	—	—	Pesticides
DEHA	bis-2-ethylhexyl adipate	Feb 2013	4.0	11	—	Plastics Chemicals
DEHP	bis-2-ethylhexyl phthalate	Feb 2013	110	42	—	Plastics Chemicals
DEHP related chemicals	mono-2-ethyl-5-carboxypentyl phthalate	Feb 2013	—	—	11	Plastics Chemicals
	mono-2-ethyl-5-hydroxyhexyl phthalate	Feb 2013	—	—	7.2	Plastics Chemicals
	mono-2-ethyl-5-oxohexyl phthalate	Feb 2013	—	—	4.6	Plastics Chemicals
	mono-2-ethylhexyl phthalate	Feb 2013	—	—	1.7	Plastics Chemicals
deltamethrin/tralomethrin		Feb 2013	not detected	—	—	Pesticides
DEP	diethyl phthalate	Feb 2013	not detected	260	—	Asthma, Fragrance Chemicals
	monoethyl phthalate	Feb 2013	—	—	3.5	Asthma, Fragrance Chemicals

CHEMICAL	FULL NAME	VISIT	DUST (µg/wipe)	AIR (ng/m³)	URINE (µg/L)	CATEGORY
diazinon		Feb 2013	not detected	—	—	Pesticides
DINP	diisononyl phthalate	Feb 2013	140	22	—	Plastics Chemicals
	mono carboxyisooctyl phthalate	Feb 2013	—	—	2.7	Plastics Chemicals
EH-TBB	2-ethylhexyl 2,3,4,5-tetrabromobenzoate	Feb 2013	—	not detected	—	Flame Retardants
fipronil		Feb 2013	not detected	—	—	Pesticides
HHCB	1,3,4,6,7,8-hexahydro-4,6,6,7,8,8-hexamethylcyclopenta-γ-2-benzopyran	Feb 2013	0.24	130	—	Asthma, Fragrance Chemicals
lindane		Feb 2013	not detected	—	—	Pesticides
mCNP	mono carboxyisononyl phthalate	Feb 2013	—	—	0.70	Plastics Chemicals
mCPP	mono-3-carboxypropyl phthalate	Feb 2013	—	—	0.60	Personal Care Product Chemicals
methoxychlor		Feb 2013	not detected	—	—	Pesticides
methyl paraben		Feb 2013	—	5.7	11	Personal Care Product Chemicals
MHiNCH	cyclohexane-1 2-dicarboxylic acid monohydroxy isononyl ester	Feb 2013	—	—	not detected	Plastics Chemicals
MiBP	mono-isobutyl phthalate	Feb 2013	—	—	12	Plastics Chemicals

© 2015 Silent Spring Institute.
This work is licensed under a Creative Commons Attribution 4.0 International License.

Appendix

CHEMICAL	FULL NAME	VISIT	DUST (µg/wipe)	AIR (ng/m³)	URINE (µg/L)	CATEGORY
MK	musk ketone	Feb 2013	—	not detected	—	Asthma, Fragrance Chemicals
MX	musk xylene	Feb 2013	—	not detected	—	Asthma, Fragrance Chemicals
naphthalene		Feb 2013	0.0089	—	—	Combustion Byproducts - PAHs
NP	4-t-nonylphenol	Feb 2013	—	19	—	Plastics Chemicals
PCB 105		Feb 2013	not detected	—	—	Banned Industrial Chemicals - PCBs
PCB 11		Feb 2013	not detected	not detected	—	Banned Industrial Chemicals - PCBs
PCB 153		Feb 2013	not detected	not detected	—	Banned Industrial Chemicals - PCBs
PCB 52		Feb 2013	not detected	not detected	—	Banned Industrial Chemicals - PCBs
PCB 95		Feb 2013	not detected	—	—	Banned Industrial Chemicals - PCBs
phenanthrene		Feb 2013	0.17	—	—	Combustion Byproducts - PAHs

CHEMICAL	FULL NAME	VISIT	DUST (µg/wipe)	AIR (ng/m³)	URINE (µg/L)	CATEGORY
piperonyl butoxide		Feb 2013	not detected	—	—	Pesticides
propoxur		Feb 2013	not detected	—	—	Pesticides
propyl paraben		Feb 2013	—	—	1.0	Personal Care Product Chemicals
pyrene		Feb 2013	0.25	—	—	Combustion Byproducts - PAHs
TBOEP	tris(2-butoxyethyl) phosphate	Feb 2013	130	not detected	—	Flame Retardants
TBOPP	tri (4-t-butoxyphenol) phosphate	Feb 2013	—	not detected	—	Flame Retardants
TBPP	tris(4-tert-butylphenyl) phosphate	Feb 2013	not detected	—	—	Flame Retardants
TCEP	tris(2-chloroethyl) phosphate	Feb 2013	not detected	not detected	—	Flame Retardants
TCIPP	tris(1-chloro-2-propyl) phosphate	Feb 2013	0.80	9.2	—	Flame Retardants
TDCIPP	tris(1,3-dichloro-isopropyl) phosphate	Feb 2013	0.13	2.6	—	Flame Retardants
TDCIPP related compound	1,3-dichloro-2-propanol	Feb 2013	—	2.9	—	Flame Retardants
TMPP	tricresyl phosphate	Feb 2013	not detected	—	—	Flame Retardants
TPHP	triphenyl phosphate	Feb 2013	0.38	not detected	—	Flame Retardants
trans-permethrin		Feb 2013	not detected	—	—	Pesticides

© 2015 Silent Spring Institute.
This work is licensed under a Creative Commons Attribution 4.0 International License.

Appendix

CHEMICAL	FULL NAME	VISIT	DUST (µg/wipe)	AIR (ng/m³)	URINE (µg/L)	CATEGORY
triclosan		Feb 2013	0.10	not detected	not detected	Asthma, Personal Care Product Chemicals
TXIB	2,2,4-trimethyl-1,3-pentanediol di-isobutyrate	Feb 2013	not detected	—	—	Plastics Chemicals

(2) Semi-structured interview guiding questions

Study Participant Report-Back Interview
Questions for Participants in Biomonitoring Studies

[Note: final interview schedules will be tailored to each collaborating study.]

Thank you for agreeing to talk with me about your participation in the Green Housing Study.

I. Participation

1. Think back to when you were first contacted to participate in the Green Housing study about exposures in your home. Why did you decide to participate in this study?
    a. What benefits, if any, did you think the study might have for you or your family personally?
    b. What benefits, if any, did you think the study might have for your neighborhood, town, or larger area in general?
2. Tell me about having a researcher come to your home to collect samples? What was that like?
3. What is your impression of what the researchers were looking for in this study?
4. When the researchers took samples, what was that experience like? [*probe*: What did you like or dislike about the experience?]

II. Interpreting and Understanding Individual Results

OK. Next I want to ask about when you received the results for your home.

5. What were your thoughts or feelings when you first learned your personal results?
6. Tell me about the way that your results were reported to you? Did the researchers contact you to ask if you wanted your results or did you contact them?
7. Please describe the information you received.
   Probe: How was this information presented to you? How useful was the particular format in which the results were reported to you? Was the language easy to understand? Were the graphics or figures easy to understand? If material was useful and /or understandable, what made it so? If anything was not useful or easy to understand, what would have made it more useful or understandable?
8. Take another look at some of the graphics that were used < *show graphics* >. What was useful or not in these graphics?
9. What do you recall about what the study found in the samples from your child and your home? [*as needed*: …the results for your child's urine; the results for your air and dust]
    a. Do you remember any specific chemicals or types of chemicals that were found in your sample?
    [*probe for names or chemical classes or sources, if they can remember them.*]
    b. Would you say that the levels of any chemicals found in your sample were "high"?
    Probe: What made you think levels were high?

c. Were you surprised by any of the results? *If so, probe*: What was surprising?

10. Did you look at any charts or pictures that showed you how your results compared to other people in the study? If yes, how useful was this information to help you understand your results?
11. Take another look at some of the data t-shirts that were used < *show graphics* >. What was understandable or not in these t-shirts? What was useful or not in these t-shirts.
12. Have you or your family members worn the data t-shirts since receiving them? *If so, probe:* a. Who wore them? b. Where were they worn? c. Did anyone ever talk with you about them? d. *If so,* what did you discuss?
13. After you got your study results, did you speak in person or over the phone with a researcher about your results? *If so, probe*:
    a. Please tell me about that. Was that conversation useful?
    b. Had you received something written first? Did you read it? What was useful or not useful about the written and graphic material you received?
14. Did you speak with a physician, nurse, or public health professional (someone not affiliated with the study)? *If so, probe*:
    a. Please tell me about that. What questions did you ask the health professional about your results?
    b. Was that useful? If not useful, what are your expectations of health care providers in this subject area?
15. Have you discussed the results with anyone else? *If so, probe*:
    a. With whom?
    b. What did you discuss?
    If they say no, gently probe: perhaps your < *(if appropriate) husband / wife, one of your children* >, another family member, a neighbor, or friend…?
16. What do you think the results tell you about your health or the health of other family members?
    a. …about past health issues that have come up in your family?
    b. …about the future health of your family?
17. Are you glad to have learned about the sampling results for your own home?
    Probe: Why? Why not?
18. How useful was learning your results? What do you think you can do about the chemicals that were found in your blood or urine?

III. Reading & Interpreting General/Study Results

So far, we've talked about what the study found in your <*sample*>. Now let's talk about what the study found overall.

19. Did you learn about the results for the study as a whole? *Probe:*
    a. What was or was not useful about the way the study results were reported?
    b. Are there results for specific chemicals that you think are important?
    c. Were you surprised by anything in the results? *If yes,* What was surprising?
20. If you were asked to describe to family, friends, and neighbors what you learned from the study, what would you tell them?

[ASK QUESTION 21-22 IF INTERVIEWS TAKE PLACE AFTER A PUBLIC MEETING OR NEWS MEDIA COVERAGE. OTHERWISE, THEY WILL BE OMITTED.]

21. Did you attend any community meetings about the study?
    a. Was this a useful format for you to get information about the study? Why or why not?
22. How else did you hear about the overall study results?
    a. Through the television, radio, or newspapers?
        If so, probe: Which ones?
    b. From a community organization?
        If so, probe: Which ones?

IV. General Implications of Study

23. Did the study change your thinking about sources of chemical exposures for yourself and your family, or how you or your family get exposed to different chemicals?
    If yes, probe:
    a. Can you tell me how it changed your thinking?
24. Did the study results change your thinking about sources of chemical exposures in your community?
    If yes, probe:
    a. Can you tell me how it changed your thinking?
25. Have you considered making any changes to reduce chemical exposures as a result of the study?
    If yes, probe:
    a. Can you tell me about any changes you have made or are planning to make?

    If no, probe:
    b. Are there changes that you would like to make, but aren't sure how? [If yes, probe: what kinds of changes?]
    c. Have you considered community actions to reduce chemical exposures as a result of the study? [If yes, probe: what kinds of actions?]

26. Did the study change your views about what role you think chemicals might play in disease or illness? *If so, probe:*
    a. In what ways?
27. As a result of this study, are there any changes that you would like to see in how companies use chemicals in their products? *If so, probe:*
    a. What changes would you like to see?
28. As a result of this study, are there any changes that you would like to see in how the government deals with chemicals and consumer products? *If so, probe:*
    a. What changes would you like to see?
29. What additional information would you like to have as a result of this study?
30. In terms of what you learned from this study, what part of the study was most useful for you?

V. General Implications for Report-Back

31. Based on your experience with this study, and thinking about similar studies in the future, do you think that researchers should offer study participants their own personal results?
    Probe: Why or why not?
32. Would you participate in another study like this again? Why or why not?
33. What advice do you have for researchers about reporting results in future studies like this one? *Probe*: Do you have specific suggestions for how they might improve how they report this information in the future?

VI. Final Thoughts

34. Is there anything else you'd like to comment on?

Thank you very much for your time and thoughtful participation in this study. Your input will be important for future follow-up on the results in this study and in other chemical exposure studies.
